# Porous aromatic framework with mesopores as a platform for a super-efficient heterogeneous Pd-based organometallic catalysis†
†Electronic supplementary information (ESI) available: Experimental details including synthesis and experimental methods. See DOI: 10.1039/c8sc00510a


**DOI:** 10.1039/c8sc00510a

**Published:** 2018-03-02

**Authors:** Li-Ping Jing, Jin-Shi Sun, Fuxing Sun, Peng Chen, Guangshan Zhu

**Affiliations:** a State Key Laboratory of Inorganic Synthesis and Preparative Chemistry , College of Chemistry , Jilin University , 2699 Qianjin Street , Changchun 130012 , China . Email: pengchen@jlu.edu.cn; b Key Laboratory of Polyoxometalate Science of the Ministry of Education , Faculty of Chemistry , Northeast Normal University , Changchun 130024 , China . Email: zhugs100@nenu.edu.cn

## Abstract

A strategy using a mesoporous amine-tagged porous aromatic framework (**PAF70-NH_2_**) to immobilize a palladium (Pd)-based molecular catalyst has been developed. The resulting immobilized catalyst **PAF70-Pd**, in which the framework is entirely constructed by phenyl rings linked with stable carbon–carbon bonds, has high structural rigidity and stability. Compared with the known porous organic material immobilized Pd-based catalysts, **PAF70-Pd** has the highest Pd content so far. Moreover, **PAF70-Pd** has extremely high catalytic activity with good size selectivity and very easy recyclability in catalyzing the Suzuki–Miyaura coupling reaction. In the current system, the catalyst loading could be as low as 0.001 mol% and the TOF value could go up to 28 800 h^–1^ which is far higher than those of the known porous organic material immobilized Pd-based catalysts. In order to elucidate the particularly high catalytic efficiency of **PAF70-Pd**, we prepared **PAF1-Pd** from **PAF1-NH_2_** for comparison. **PAF1-Pd** has a higher Pd content than **PAF70-Pd**. However, due to the absence of large enough mesopores in **PAF1-NH_2_**, **PAF1-Pd** has almost no catalytic activity under the same conditions, which definitely demonstrated that the intrinsic mesoporosity of **PAF70-NH_2_** plays a crucial role in the superb catalytic efficiency of **PAF70-Pd**. This strategy to immobilize Pd-based molecular catalysts has very good expansibility to be applied in the immobilization of different organometallic catalysts into the pores of PAFs, which also has very high potential in the chemical and pharmaceutical industry.

## Abstract 2

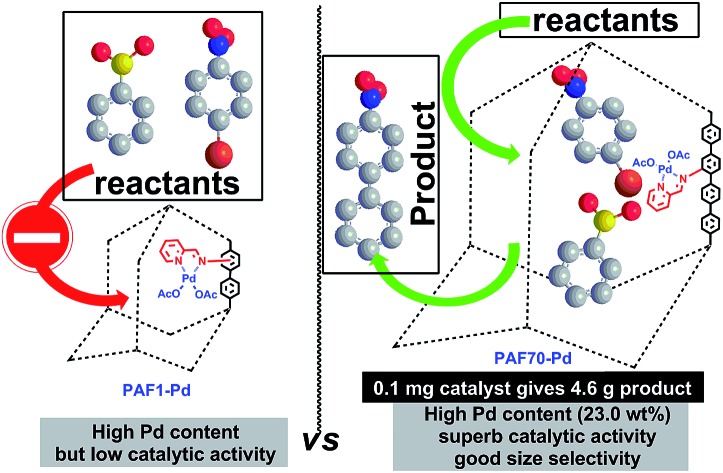
A porous aromatic framework with mesopores was used as a platform for an immobilized Pd catalyst with superb catalytic activity and size selectivity for the Suzuki–Miyaura coupling reaction.

## 1. Introduction

Organometallic catalysis, which uses a metal-based molecular catalyst to catalyze an organic reaction, is a very important field of organic chemistry. The excellent catalytic activity of the organometallic catalysts (metal-based molecular catalysts) has attracted intensive interest from chemists in diverse research fields. Thereinto, palladium (Pd)-based organometallic catalysts are versatile tools that can catalyze various organic reactions such as the Suzuki–Miyaura coupling reaction and Heck reaction.^1^–^3^ However, the high price of Pd-based catalysts and the Pd-residue in the products have greatly limited their applications in academia and industry. Immobilization of Pd-based catalysts onto some supported solid materials such as activated carbon, inert inorganic zeolites or organic polymers, typically by physical adsorption or chemical grafting (binding), is a good method to solve the above problems.^4^–^9^ Although the rapid development of immobilization of Pd-based catalysts has been achieved, this field still suffers from reduced catalytic activity caused by poor accessibility or low metal loadings.^10^–^29^

Using porous materials such as metal–organic frameworks (MOFs), covalent organic frameworks (COFs) or porous organic polymers (POPs) as supported materials began to appear in the last few decades, which is a good idea because of their porosity and high surface area.^6^,^7^,^30^–^36^ However, the intrinsic instability of MOFs and COFs or the flexibility of the frameworks of POPs makes this related research field still face many difficulties. In 2009, a new type of porous organic material with robust regular frameworks constructed entirely from rigid aromatic building blocks linked by stable covalent bonds, named porous aromatic frameworks (PAFs), was developed by our group and achieved intensive interest from researchers in diverse fields due to their wide range of structures and potential applications in gas sorption,^37^–^45^ separation,^46^,^47^ catalysis,^48^–^54^ *etc.* Owing to their robust structure together with high stability in most organic solvents, PAFs are extremely suitable platforms for the catalysis of organic reactions. It’s worth noting that, due to the presence of the Pd center and organic ligand, Pd-based organometallic catalysts usually have relatively large sizes. Hence, immobilization of Pd-based molecular catalysts into the porous materials often needs a large enough pore size. Most of the reported porous organic material immobilized Pd-based catalysts always suffer from low Pd utilization efficiency which might be due to that the pore space after introduction of the Pd-based catalyst is too small to accommodate the catalytic reaction. Apparently, for application of PAFs as the platforms for Pd-based organometallic catalysts, PAFs with large enough mesopores are needed. However, the synthesis of narrowly distributed mesoporous PAFs is still a challenge because of the interpenetration while using large-size monomers. Thus using PAFs as the platforms for covalent anchoring of organometallic catalysts into the pores still remains rare up to now. In this paper, we will make an attempt in this area.

Considering the need for large enough pore space for accommodating Pd-based molecular catalysts and the subsequent catalysis, in this paper, **PAF70-NH_2_**, an amine-tagged PAF with narrowly distributed mesopores which was recently reported by our group,^55^ was selected as the platform for Pd-based organometallic catalysis. In this paper, a strategy involving two post-synthesis modification steps for the introduction of the Pd-based organometallic catalyst into the pores of **PAF70-NH_2_** was used for the synthesis of our desired material, and the catalytic performance of the desired material (**PAF70-Pd**) was systematically studied. In order to further demonstrate the importance of the mesopores in **PAF70-NH_2_**, another amine-tagged PAF (**PAF1-NH_2_**) without mesopores was also used as a platform to immobilize the same Pd-based molecular catalyst, affording **PAF1-Pd** for comparison with **PAF70-Pd**.

## 2. Results and discussion

### 2.1 Synthesis of the materials

**Scheme 1:**
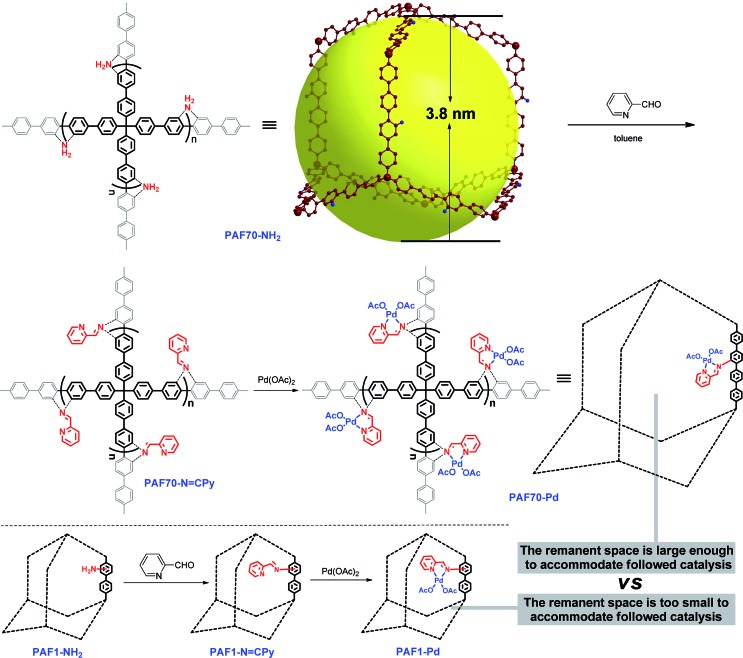
Synthetic route to **PAF70-Pd** and **PAF1-Pd**.

Firstly, as shown in Scheme 1, *via* the pre-modification procedure used in our previous literature report,^55^ we synthesized **PAF70-NH_2_**, which contains mesopores with 3.8 nm diameter and amine anchors in the pores. Then, *via* the amine anchors using a condensation reaction with picolinaldehyde, the chelating ligand unit for Pd was introduced into the material, yielding the PAF which was named **PAF70-N=CPy**. After a second post-treatment of **PAF70-N=CPy** with palladium acetate, the PAF material containing the Pd-based molecular catalyst was obtained, which was named **PAF70-Pd**. It’s worth noting that the *N*,*N*-bidentate ligand is one of the most versatile coordination systems in organometallic catalysis, which can coordinate with various metal ions and has been widely used in homogeneous catalysis. In addition, the post-synthesis modification was very facile and efficient. The above features of our synthetic method could expand the application value of the PAF material. In addition, for the purpose of comparison, we prepared **PAF1-NH_2_** according to the literature report,^51^ and using a similar two-step post-synthesis method **PAF1-Pd** (the counterpart of **PAF70-Pd**) was prepared (Scheme 1).

### 2.2 Characterization of the materials

**Fig. 1:**
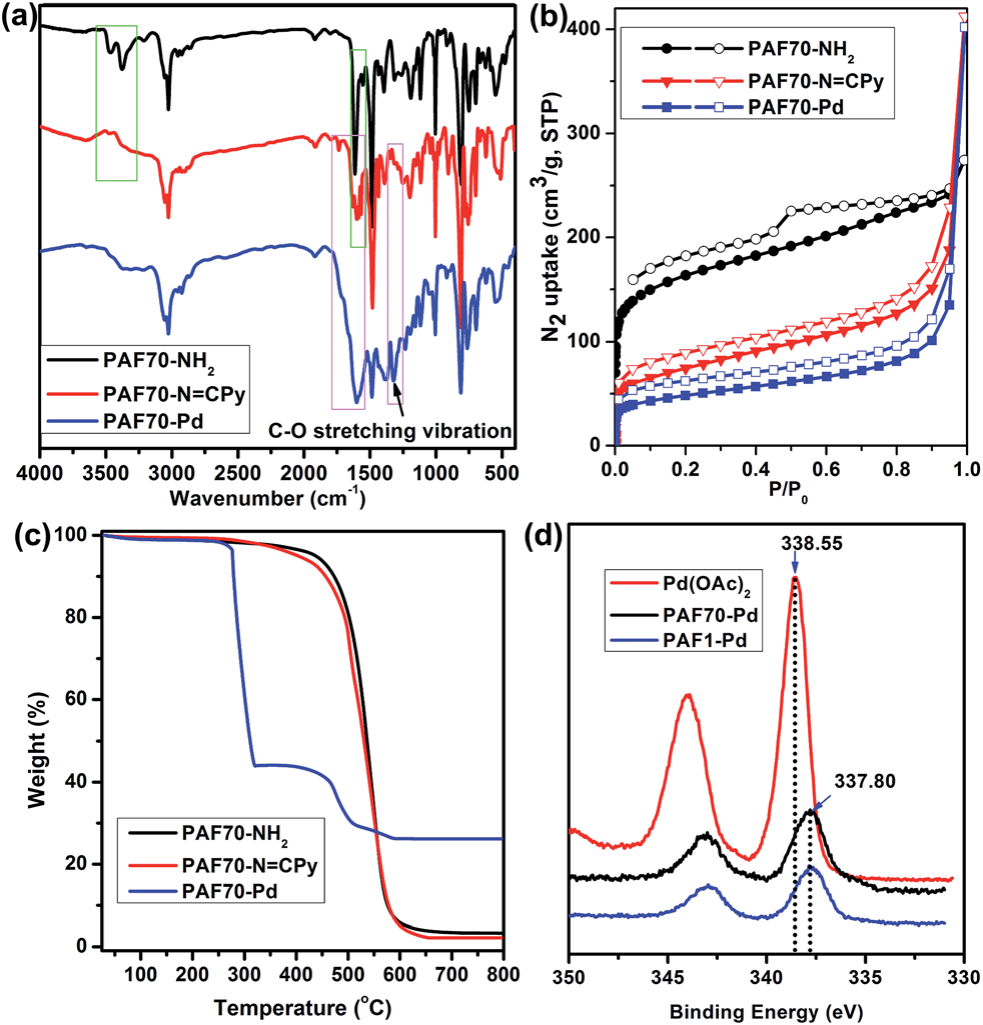
FT-IR spectra (a), nitrogen adsorption (solid symbols)–desorption (open symbols) isotherms measured at 77 K (b), and TGA plots (c) of **PAF70-NH_2_**, **PAF70-N=CPy** and **PAF70-Pd**. XPS spectra (d) of free Pd(OAc)_2_,**PAF70-Pd** and **PAF1-Pd**.

As shown in Fig. 1a, compared with the (Fourier transform infrared) FT-IR spectrum of **PAF70-NH_2_**, the strong attenuation of the characteristic double peaks of –NH_2_ (3464 and 3377 cm^–1^) and the appearance of the characteristic peaks of the Schiff base (the new peaks at around 1600 cm^–1^) in the FT-IR spectrum of **PAF70-N=CPy** indicated the formation of an imine bond and thus the successful construction of **PAF70-N=CPy**. In comparison with **PAF70-N=CPy**, in the FT-IR spectrum (Fig. 1a) of **PAF70-Pd**, the appearance and enhancement of the bands in the 1520–1750 cm^–1^ region could be attributed to the C=O stretching vibration and the new peaks at 1321 cm^–1^ could be attributed to the C–O stretching vibration, which obviously indicated that **PAF70-Pd** was successfully obtained through our strategy. In addition, the successful synthesis of **PAF1-Pd** was also confirmed through a similar analysis of the FT-IR spectra (more details could be found in Fig. S15 in the ESI†).

Nitrogen adsorption–desorption isotherms for the obtained materials were measured at 77 K. As shown in Fig. 1b, at low relative pressures, **PAF70-NH_2_**, **PAF70-N=CPy** and **PAF70-Pd** all showed sharp uptakes, indicating the existence of micropores in the materials. It’s worth noting that, in the desorption branch of **PAF70-NH_2_**, a relatively sharp hysteresis demonstrated the presence of narrowly distributed mesopores. Compared with **PAF70-NH_2_**, the corresponding hysteresis disappeared in the desorption branches of **PAF70-N=CPy** and **PAF70-Pd**, which indicated the disappearance of the mesopores after post-modification of **PAF70-NH_2_**. The apparent surface area calculated from the Brunauer–Emmett–Teller (BET) model was 599 m^2^ g^–1^ for **PAF70-NH_2_**, 263 m^2^ g^–1^ for **PAF70-N=CPy**, and 172 m^2^ g^–1^ for **PAF70-Pd**. Through the change of pore size distributions calculated by non-local density functional theory (NLDFT), it was clear that the mesopores with a pore width of 3.8 nm of **PAF70-NH_2_** disappeared in **PAF70-Pd** (see Fig. S10 in the ESI†). The decrease of the BET surface area and the disappearance of mesopores from **PAF70-NH_2_** to **PAF70-Pd** further proved the successful introduction of the Pd-based functional groups into the pores of the PAF.

Thermogravimetric analysis (TGA) was performed to test the thermal stabilities of the above PAF materials. As shown in Fig. 1c, **PAF70-NH_2_** (black curve) and **PAF70-N=CPy** (red curve) showed similar TGA curves. There is almost no weight loss before 300°C, which suggested the high thermal stability of **PAF70-NH_2_** and **PAF70-N=CPy**. At about 400°C, the framework decomposition started and when the temperature was above 500°C the decomposition became obvious. The 3.96 wt% residue for **PAF70-NH_2_** and 2.15 wt% residue for **PAF70-N=CPy** at 800°C could be ascribed to some palladium oxide residue which originated from the Pd catalysts in the preparation process of **PAF70-NH_2_**. As shown in Fig. 1c, **PAF70-Pd** (blue curve) had a 56% weight loss at 277–320°C. This weight loss could be attributed to the decomposition of both *N*,*N*-bidentate ligand and AcO^–^ species which were directly connected to the Pd center. Compared with **PAF70-N=CPy**, **PAF70-Pd** showed lower stability, which might be due to that the Pd species could catalyze the cleavage of carbon–carbon bonds around the Pd centers in the PAF material.^56^ After a further obvious decomposition of the framework that started at 450°C, there was a 26.2 wt% palladium oxide residue left at 800°C. In addition, all the three PAF materials could not be dissolved or decomposed in almost all common solvents such as water, ethanol, dichloromethane, toluene, tetrahydrofuran, ethyl acetate, hexane, diethyl ether, *etc.* The high thermal stability and chemical stability made **PAF70-Pd** fully satisfy the demands of catalysis. The TGA analysis of **PAF1-Pd** can be found in the ESI (Fig. S17†). The Pd content was further determined by inductively coupled plasma (ICP) analysis, which revealed that 23.0 wt% of Pd was incorporated into **PAF70-Pd** and 25.1 wt% of Pd was incorporated into **PAF1-Pd**. These were in agreement with the TGA analysis. Importantly, to the best of our knowledge, **PAF70-Pd** and **PAF1-Pd** have higher Pd contents than other reported porous organic material immobilized Pd catalysts, which significantly profits from that the pores of our PAF materials could endow high surface area for immobilizing the Pd coordination system.

In order to further investigate the incorporation of palladium within **PAF70-N=CPy** and **PAF1-N=CPy**, X-ray photoelectron spectroscopy (XPS) was performed. As shown in Fig. 1d, the binding energy (BE) at 337.80 eV, assigned to the Pd3d_5/2_ orbital, indicated that the Pd species in **PAF70-Pd** and **PAF1-Pd** are present in the +2 state. Compared with the BE of 338.55 eV for free Pd(OAc)_2_, the BE for Pd species in **PAF70-Pd** and **PAF1-Pd** negatively shifted by 0.75 eV. This negative shift indicated that Pd(OAc)_2_ has strong coordination with the *N*,*N*-bidentate ligand in **PAF70-N=CPy** and **PAF1-N=CPy**.^11^,^26^

**Fig. 2:**
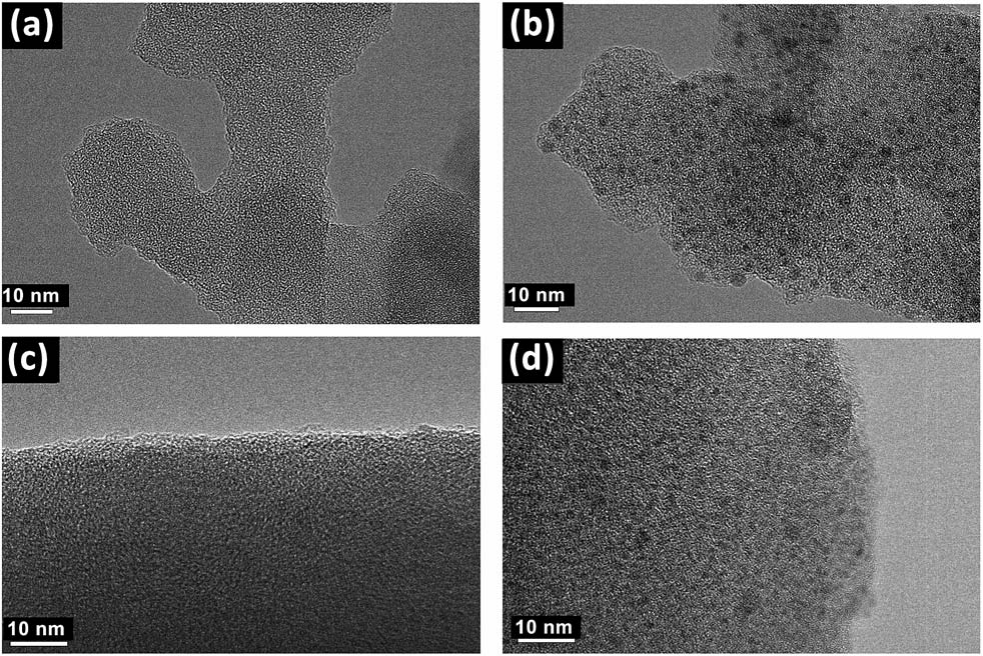
TEM images of **PAF70-N=CPy** (a), **PAF70-Pd** (b), **PAF1-N=CPy** (c) and **PAF1-Pd** (d).

Transmission electron microscopys (TEM) images obviously showed the successful introduction of Pd species into the PAF materials. As shown in Fig. 2a and Fig. 2b, compared with **PAF70-N=CPy**, some evenly distributed black dots with a mean diameter of about 1 nm emerged in the TEM images of **PAF70-Pd**, indicating that the Pd species are uniformly dispersed in the frameworks of the PAF material, which was in accordance with the above analysis of the TGA curve of **PAF70-Pd**. Similarly, compared with **PAF1-N=CPy** (Fig. 2c), the TEM image of **PAF1-Pd** (Fig. 2d) also showed uniformly dispersed Pd species. This demonstrated that the Pd-based catalyst could also be anchored into the pores of the **PAF1-NH_2_** material.

### 2.3 Catalytic performance of **PAF70-Pd**

After confirming the formation of the desired **PAF70-Pd** and **PAF1-Pd**, their catalytic properties were then studied. Suzuki–Miyaura coupling reaction, one of the representative Pd-catalyzed reactions, was selected as the model reaction to study their catalytic performance. Firstly, some control experiments were conducted using *p*-bromonitrobenzene and phenylboronic acid as model substrates and **PAF70-Pd** as the catalyst. As shown in entries 1–3 of Table 1, among the screened solvents (CH_2_Cl_2_, *p*-xylene and EtOH), EtOH gave the best results in terms of the reaction rate and yield of the current catalytic Suzuki–Miyaura coupling reaction. Increasing the reaction temperature from 25°C to 80°C improved the reaction rate significantly (Table 1, entries 3–6). The catalyst loading screening showed that increasing the catalyst loading could improve the reaction rate (Table 1, entries 6–9). It was exciting to note that when the catalyst loading of **PAF70-Pd** was reduced to 0.01 mol%, the current catalytic reaction could still occur rapidly (Table 1, entry 8) and when the catalyst loading of **PAF70-Pd** was reduced to 0.001 mol%, the current catalytic reaction could still occur smoothly (Table 1, entry 9). Under the best conditions (Table 1, entry 8) obtained from the above screenings, the current reaction could not occur without a catalyst (Table 1, entry 10). In addition, **PAF70-N=CPy** (Table 1, entry 11) with the palladium residue in the material could not catalyze the reaction, indicating that the Pd residue from the preparation process in these materials has no catalytic activity. The above results demonstrated that **PAF70-Pd** is indeed an efficient catalyst for the Suzuki–Miyaura coupling reaction. Furthermore, the supernatant liquid of the EtOH suspension of **PAF70-Pd** showed no catalytic activity for the coupling reaction (Table 1, entry 12) even in a much longer time, which indicated no leakage of catalytically active species from the **PAF70-Pd** catalyst during the catalysis process. Thus the current **PAF70-Pd** catalyzed reaction proceeds *via* a heterogeneous catalytic process.

**Table 1:** The control experiments for **PAF70-Pd** catalyzed Suzuki–Miyaura coupling reaction^*a*^

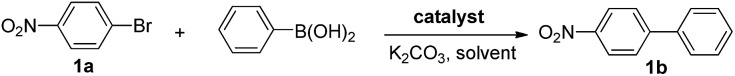					
Entry	Catalyst (catalyst loading)	Solvent	*T* [°C]	Time	Yield^*b*^
1	**PAF70-Pd** (0.5 mol%)	CH_2_Cl_2_	40	12h	0
2	**PAF70-Pd** (0.5 mol%)	*p*-Xylene	150	4h	92%
3	**PAF70-Pd** (0.5 mol%)	EtOH	25	1h	92%
4	**PAF70-Pd** (0.5 mol%)	EtOH	40	45min	96%
5	**PAF70-Pd** (0.5 mol%)	EtOH	60	20min	95%
6	**PAF70-Pd** (0.5 mol%)	EtOH	80	7min	97%
7^*c*^	**PAF70-Pd** (0.1mol%)	EtOH	80	15min	96%
8^*c*^	**PAF70-Pd** (0.01 mol%)	EtOH	80	25min	97%
9^*d*^	**PAF70-Pd** (0.001 mol%)	EtOH	80	4h	93%
10	No catalyst	EtOH	80	12h	0
11	**PAF70-N=CPy**	EtOH	80	12h	0
12^*e*^	The supernatant liquid of the EtOH suspension of **PAF70-Pd** and K_2_CO_3_	EtOH	80	12h	0
13	**PAF1-Pd** (0.01 mol%)	EtOH	80	25min	<5%

^*a*^Reaction conditions (unless otherwise noted): a solution of **1a** (0.5 mmol), phenylboronic acid (0.75 mmol), K_2_CO_3_ (1.0 mmol), and the catalysts (for entry 10, no catalyst was added; for entry 11, 1.2 mg **PAF70-N=CPy** was added as the catalyst; for other entries, the catalysts were added at the indicated loadings based on Pd) in 2 mL of solvent was stirred at the indicated temperature for the indicated time.

^*b*^The isolated yield.

^*c*^The reaction scale was 2.5 mmol of **1a**.

^*d*^The reaction scale was 25.0 mmol of **1a**.

^*e*^1.2 mg **PAF70-Pd** and K_2_CO_3_ (1.0 mmol) were immersed in 2 mL of EtOH for 2 h at 80°C; after centrifugation, to the supernatant liquid were added 0.5 mmol **1a**, 0.75 mmol phenylboronic acid and 1.0 mmol K_2_CO_3_, then the resulting mixture was stirred at 80°C for 12 h.

For comparison, **PAF1-Pd** was then employed as the catalyst for the current Suzuki–Miyaura coupling reaction under the best conditions as shown in entry 8 of Table 1. Compared with **PAF70-Pd** which gave a 97% yield (Table 1, entry 8), **PAF1-Pd** showed almost no catalytic activity (<5% yield, Table 1, entry 13) under the same conditions which should be due to that the remnant space in the pores after introducing the Pd-catalyst was too small to accommodate the current catalysis. This comparison fully demonstrated the importance of the large enough mesopores in **PAF70-NH_2_** for its application in immobilizing large-size metal-based molecular catalysts.

The catalytic performance of **PAF70-Pd** was further tested using a series of aryl bromides as the reaction substrates at a 0.01 mol% catalyst loading. As shown in Table 2, bromobenzene **9a** (entry 9) or the substituted aryl bromides with either an electron-withdrawing group (such as –NO_2_, CHO, –Br, –F and –CN, entries 1–5) or an electron-donating-group (such as –OMe, –Me and –(OH)CHCH_3_, entries 6–8) afforded the cross-coupling products in excellent yields (up to >99%) with high turnover frequency (TOF) values (all ≥14 700 h^–1^), demonstrating the wide generality and functional tolerance of the current system.

**Table 2:** **PAF70-Pd** catalyzed Suzuki–Miyaura coupling reaction^*a*^

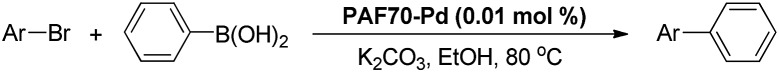					
Entry	Ar–Br	Product	Time (min)	Yield^*b*^	TOF (h^–1^)^*c*^
1	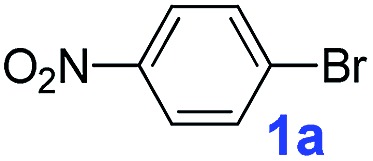	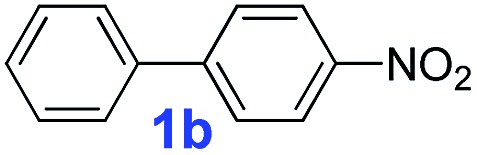	25	97%	23 280
2	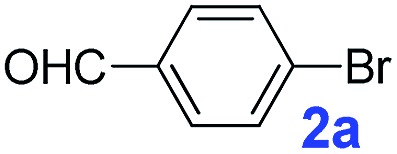	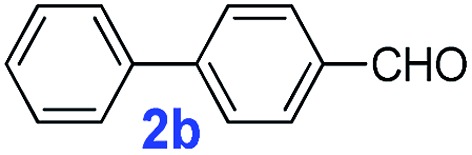	30	96%	18 432
3	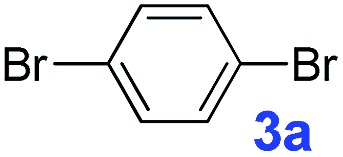	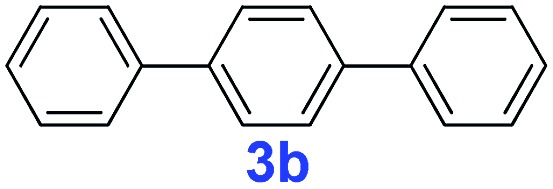	30	95%	19 000
4	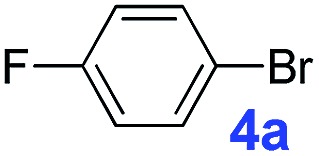	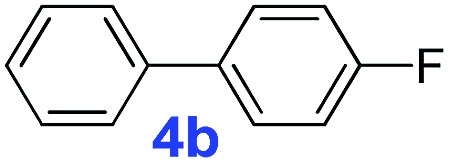	30	90%	18 000
5	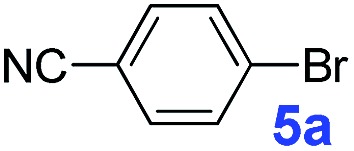	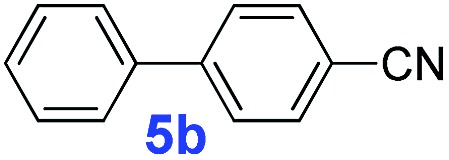	40	>99%	>14 850
6	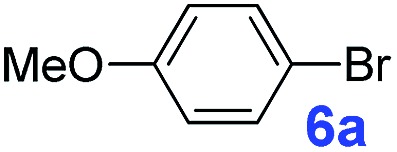	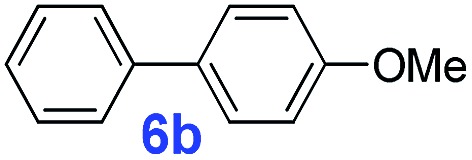	40	98%	14 700
7	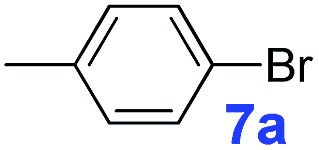	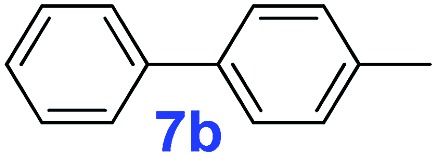	35	92%	15 771
8	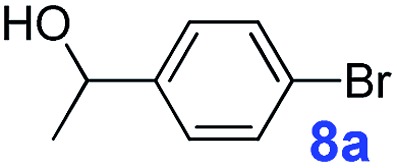	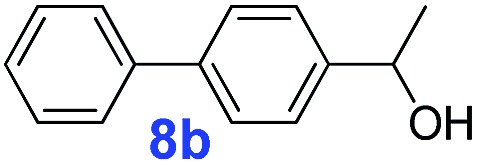	40	>99%	>14 850
9	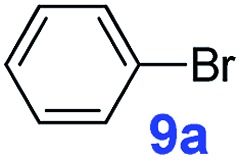	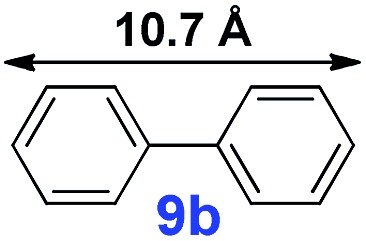	20	96%	28 800
10	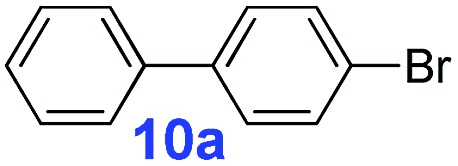	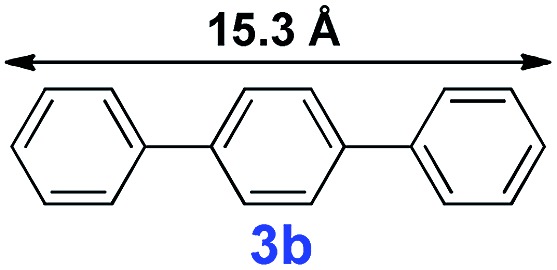	20	35%	—
11^*d*^	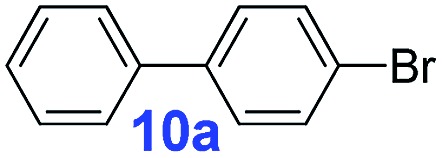	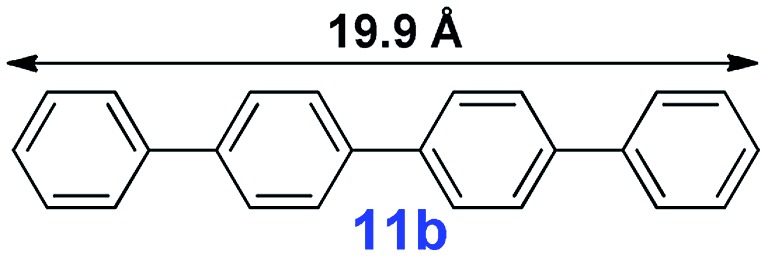	20	<5%	—

^*a*^Reaction conditions: a solution of 2.5 mmol **1a**, 3.75 mmol phenylboronic acid, 5.0 mmol K_2_CO_3_ and **PAF70-Pd** (0.01 mol%) in 10 mL of EtOH was stirred at 80°C for the indicated time.

^*b*^The isolated yield.

^*c*^TOF = (moles of product)/(moles of Pd in the catalyst × reaction time).

^*d*^For entry 11, 4-biphenylboronic acid was used instead of phenylboronic acid.

For the porous material immobilized catalysts with narrowly distributed pore size, the size selectivity is a signature feature of the catalyzed reaction which could occur in the pores. In order to investigate the size selectivity of **PAF70-Pd**, some contrast tests were performed as shown in entries 9–11 of Table 2. Compared with **9a**, which could smoothly transform to **9b** completely (Table 2, entry 9), the larger-size **10a** reacted more slowly under the same conditions in the same time (35% yield, Table 2, entry 10). When **10a** reacted with the larger-size 4-biphenylboronic acid, the reaction rate further decreased and even almost no product was obtained under the same conditions in the same time (Table 2, entry 11). The above size selectivity obviously indicated that the catalytic reaction could occur inside the pores of **PAF70-Pd**.

For heterogeneous catalysts, recyclability is an important factor. Hence the recyclability of **PAF70-Pd** as the catalyst was tested by subjecting it to 3 cycles of the Suzuki–Miyaura coupling reaction of 4-bromonitrobenzene **1a** and phenylboronic acid (Table S1 in the ESI†). After each cycle, **PAF70-Pd** was easily recovered by centrifugation followed by washing and could be directly used in the next cycle for cycles 2–3, in which the substrate dosages were the same as that in cycle 1. After 3 cycles, the recovered **PAF70-Pd** was dried *in vacuo* at 120°C for 18 h. ICP analysis showed that the recovered **PAF70-Pd** had 22.7 wt% of Pd content, which had no obvious change compared with the fresh **PAF70-Pd** (23.0 wt% of Pd content). These indicated that there is very low metal leaching during the reaction process. The results demonstrated that **PAF70-Pd** could undergo at least 3 cycles of the reaction without obvious loss of catalytic activity.

### 2.4 Comparison with the previously reported porous organic material (POPs and COFs) immobilized Pd catalysts

**Fig. 3:**
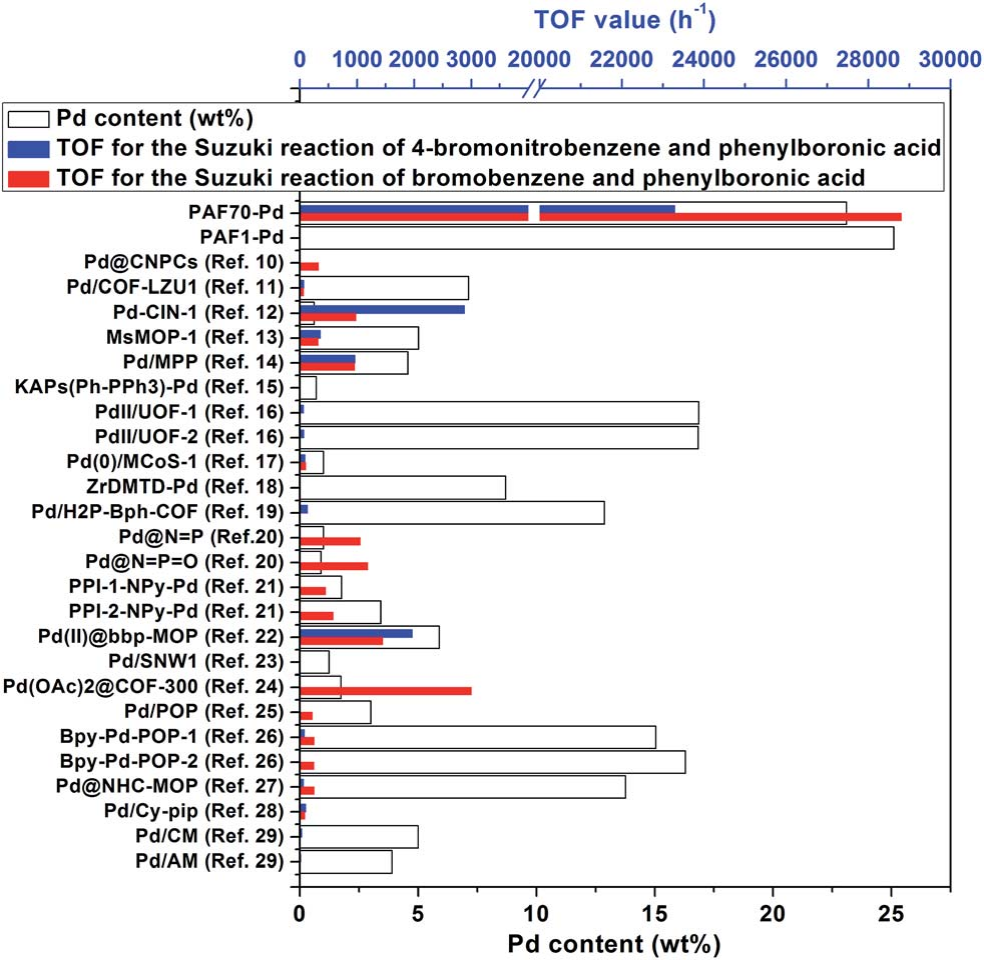
Pd contents and the catalytic performances in the Suzuki–Miyaura coupling reaction of **PAF70-Pd**, **PAF1-Pd** and other reported porous organic material (POPs and COFs) immobilized Pd catalysts.

Data on the Pd contents and the TOF values of corresponding catalytic systems of **PAF70-Pd**, **PAF1-Pd** and previously reported porous organic material immobilized Pd catalysts are given in Fig. 3 and Table S2 in the ESI.† The TOF values were all calculated for the whole reaction process after complete conversion of the reactant under the respective optimum conditions. It is quite noticeable that **PAF70-Pd** and **PAF1-Pd** have the highest Pd contents, which might be due to the high effective surface areas of our materials. Moreover, **PAF70-Pd** showed far higher TOF values than other catalysts in Fig. 3. In addition, **PAF70-Pd** gave a rare example of a catalytic system with size selectivity in this field (Table S2 in the ESI†). These excellent catalytic properties of **PAF70-Pd** could be attributed to the large enough remnant pore space after the introduction of the catalytic sites. That is, the remnant pore space could accommodate the reactants entering into the pores and the products exiting outside of the pores.

## 3. Conclusions

Based on the mesoporous PAF (**PAF70-NH_2_**), by a post-synthesis method, **PAF70-N=CPy** with an *N*,*N*-bidentate ligand was successfully obtained. After a second post-treatment with palladium acetate, the PAF material containing the Pd-based molecular catalyst, named **PAF70-Pd**, was prepared. Because the narrowly distributed mesopores (3.8 nm) in **PAF70-NH_2_** endow sufficient pore space for immobilizing the Pd-based molecular catalyst with a relatively large size, the resulting immobilized catalyst **PAF70-Pd** has evenly distributed Pd species and high Pd content (23.0 wt%). Furthermore, **PAF70-Pd** showed superb catalytic activity for catalyzing the Suzuki–Miyaura coupling reaction with good size selectivity and very easy recyclability. Compared with the reported porous organic material immobilized Pd catalysts, **PAF70-Pd** has the highest Pd content and exhibits a far higher TOF value when catalyzing the same Suzuki–Miyaura coupling reaction. By comparison with **PAF1-Pd**, it was clearly demonstrated that the mesopores in **PAF70-NH_2_** are very important for the high activity of **PAF70-Pd**. After the introduction of Pd species with a relatively large size, **PAF70-Pd** still has large enough pore space to accommodate the catalyzed reaction. This could significantly enhance the utilization efficiency of the Pd catalyst in the material. Our strategy supplied a versatile method for the immobilization of different organometallic catalysts into the pores of PAFs, which will promote the development of PAF-based organometallic catalysts.

## Conflicts of interest

There are no conflicts to declare.

## Supplementary Material

Supplementary information
