# Supplementary material for: Porous aromatic framework with mesopores as a platform for a super-efficient heterogeneous Pd-based organometallic catalysis
Source: Chem Sci. 2018 Mar 2;9(14):3523–30. doi: 10.1039/c8sc00510a (PMC5934748; doi:10.1039/c8sc00510a)
Supplement: Supplementary file 1 [file SC-009-C8SC00510A-s001.pdf]

Electronic Supplementary Material (ESI) for Chemical Science.
This journal is © The Royal Society of Chemistry 2018

Supplementary Information

Porous aromatic framework with mesopores as a platform for a super-efficient heterogeneous Pd-based organometallic catalysis

Li-Ping Jing, Jin-Shi Sun, Fuxing Sun, Peng Chen*, and Guangshan Zhu*

TABLE OF CONTENT:

General........ S1
1. Synthesis of The Monomers of **PAF70-Pd** S1
2. Synthesis of **PAF70-NH$_2$**........ S2
3. Synthesis of **PAF70-N=CPy** S4
4. Synthesis of **PAF70-Pd**........ S6
5. Synthesis of **PAF1-Pd**........ S8
6. **PAF70-Pd** Catalyzed Suzuki-Miyaura Coupling Reaction S12
7. Recyclability of **PAF70-Pd** for Catalyzing Suzuki-Miyaura Coupling Reaction S13
8. Comparison with the previously reported POPs immobilized Pd catalysts........ S14
9. References........ S15
9. Copies of NMR Spectra S15

General

All moisture or oxygen-sensitive reactions were carried out under an argon atmosphere in oven or heat-dried flasks. The solvents used were purified by distillation over the drying agents indicated and were transferred under argon: Et_2O (Na), CH_2Cl_2 (CaH_2), THF (Na), EtOH (Grignard reagent). All reactions were monitored by thin-layer chromatography (TLC) on gel F_{254} plates using UV light as visualizing agent (if applicable), and a solution of ammonium molybdate tetrahydrate (50 g/L) in EtOH followed by heating as developing agents. The products were purified by flash column chromatography on silica gel (200-300 meshes) from the Qingdao Marine Chemical Factory in China.

^{1}H NMR and ^{13}C NMR spectra were recorded in $CDCl_3$ or DMSO-*d*6 solution on a Varian 300 MHz instrument (300 MHz for ^{1}H NMR, 75 MHz for ^{13}C NMR) or a Bruker 500 MHz instrument (500 MHz for ^{1}H NMR, 125 MHz for ^{13}C NMR). Chemical shifts were denoted in ppm (δ), and calibrated by using residual undeuterated solvent ($CHCl_3$ (7.26 ppm), DMSO-*d*5 (2.50 ppm) or (0.00 ppm)) as internal reference for ^{1}H NMR and the deuterated solvent ($CDCl_3$ (77.00 ppm) or DMSO-d6 (39.51 ppm)) or tetramethylsilane (0.00 ppm) as internal standard for ^{13}C NMR. Coupling constants reported in Hz constitute ^{3}J (H, H) and ^{4}J (H, H) coupling constants, unless otherwise noted. The following abbreviations were used to explain the multiplicities: s = singlet, d = doublet, t = triplet, q = quartet, br = broad, td = triple doublet, dt = double triplet, m = multiplet. High-resolution mass spectral analysis (HRMS) data were measured on Agilent 1290-microTOF Q II by means of the ESI technique. FT-IR spectra were recorded on a Bruker IFS 66v/S Fourier transform infrared spectrometer. Thermogravimetric analysis (TGA) was performed using a Netzch Sta 449c thermal analyzer system at a heating rate of 10 °C min^{-1} in an air atmosphere. The N_2 adsorption–desorption isotherms were measured on a Quantachrome Autosorb-iQ2 analyzer. Powder X-ray diffraction (PXRD) was performed by a Riguku D/MAX2550 diffractometer using Cu-Kα radiation, 40 kV, 200 mA with a scanning rate of 1° min^{-1} (2θ). Elemental analysis was carried out on a PerkinElmer 240C elemental analyzer. X-ray photoelectron spectroscopy (XPS) was performed using Thermo ESCALAB 250. Transmission electron microscopy (TEM) was recorded using a JEOL JEM 3010 instrument with an acceleration voltage of 300 kV. Scanning electron Microscopy (SEM) analysis was performed on a JEOS JSM 6700 system.

1. Synthesis of The Monomers of PAF70-Pd

■ Synthesis of the monomer **1**:

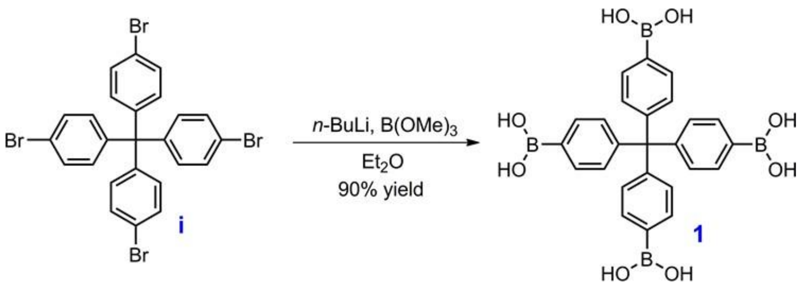

To a suspension of tetra(4-bromophenyl)methane **i** (2.0 g, 3.14 mmol) in Et_2O (150 mL) was added a solution of *n*-BuLi in hexane (2.5 M, 7.5 mL, 18.9 mmol, 6.0 equiv.) via cannula at -78 °C. The resultant reaction mixture was then allowed to warm to room temperature and stirred for 10 h. After cooling to -78 °C again, $B(OMe)_3$ (2.61 g, 25.2 mmol, 8.0 equiv.) was added to the reaction mixture, and the resultant reaction mixture was then allowed to warm to room temperature and stirred for 3 h. After removing the solvent by evaporation, to the resulting residue was added aqueous HCl solution (2.4 M, about 60 mL) and the resultant mixture was filtrated. To the resulting solid was added aqueous NaOH solution (1 M, 10 mL) and the resultant mixture was filtrated. Then the pH value of the filtrate was adjusted to <6 by adding aqueous HCl solution (1 M, about 10 mL). After filtration, the resultant solid was washed with H_2O (2 × 15 mL) and dried in vacuo to give **1** (1.41 g, 2.84 mmol, 90% yield).

1 : ^{1}H NMR (300 MHz, DMSO-*d*6): δ = 8.00 (brs, 8H), 7.68 (d, *J* = 8.4 Hz, 8H), 7.14 ppm (d, *J* = 8.4 Hz, 8H); ^{13}C NMR (75 MHz, $CDCl_3$): δ = 148.2, 133.5, 131.8, 129.6, 65.0 ppm.

■ Synthesis of the monomer **2**:

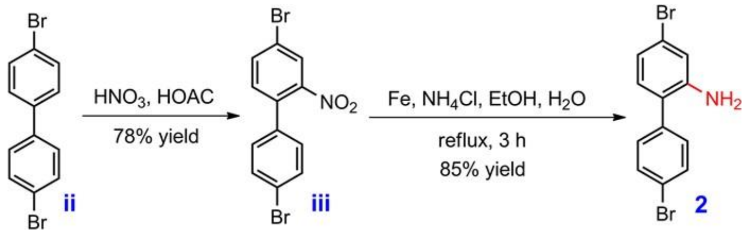

4,4'-Dibromobiphenyl **ii** (8.0 g, 25.6 mmol) was dissolved in glacial acetic acid (120 mL) at 100 °C. Then fuming nitric acid (36 mL) was added dropwise via cannula and the resultant reaction mixture was stirred at 100 °C for 2 h. Then the reaction mixture was poured into 200 mL cold water (0 °C). After filtration under reduced pressure, the resultant solid was washed with H_2O (3 × 15 mL) and dried in vacuo. The obtained crude product was dissolved in 150 mL boiling EtOH at 90 °C. The mixed solution was stood at room temperature for crystallization for 12 h. Following the filtration under reduced pressure, the solid was isolated and dried in vacuo to give **iii** (7.14 g, 20.0 mmol, 78% yield).

iii : ^{1}H NMR (300 MHz, $CDCl_3$): δ = 8.03 (d, *J* = 1.8 Hz, 1H), 7.76 (dd, *J* = 8.1, 1.8 Hz, 1H), 7.56 (d, *J* = 8.4 Hz, 2H), 7.29 (d, *J* = 8.1 Hz, 1H), 7.16 ppm (d, *J* = 8.4 Hz, 2H); ^{13}C NMR (75 MHz, $CDCl_3$): δ = 149.3, 135.5, 135.3, 134.1, 133.0, 132.0, 129.4, 127.2, 123.1, 121.8 ppm.

To **iii** (5.15 g, 14.4 mmol) obtained above was sequentially added Fe powder (2.42 g, 43.3 mmol, 3 equiv.), NH_4Cl (1.54 g, 28.8 mmol, 2 equiv.), 120 mL EtOH and 30 mL H_2O. The obtained mixture was stirred and refluxed at 90 °C for 3 h. After cooling to room temperature, the pH value of the mixture was adjusted to 7 by adding saturated aqueous $NaHCO_3$ solution (about 100 mL). Following the filtration under reduced pressure, EtOH was removed from the obtained filtrate by evaporation. The resultant residue was extracted by CH_2Cl_2 (4 × 100 mL) and the combined organic solution was dried over Na_2SO_4. After evaporation of the solvent under vacuum, the crude product was dissolved in 50 mL boiling CH_2Cl_2 at 70 °C. The mixed solution was stood at room temperature for crystallization for 12 h. Following the filtration under reduced pressure, the solid was isolated and dried in vacuo to give **2** (4.01 g, 12.3 mmol, 85% yield).

2 : ^{1}H NMR (300 MHz, $CDCl_3$): δ = 7.57 (d, *J* = 8.4 Hz, 2H), 7.29 (d, *J* = 8.4 Hz, 2H), 6.94–6.90 (m, 3H), 3.84 ppm (brs, 2H); ^{13}C NMR (75 MHz, $CDCl_3$): δ = 144.7, 137.3, 132.1, 131.5, 130.6, 125.1, 122.4, 121.60, 121.56, 118.2 ppm.

2. Synthesis of PAF70-NH_2

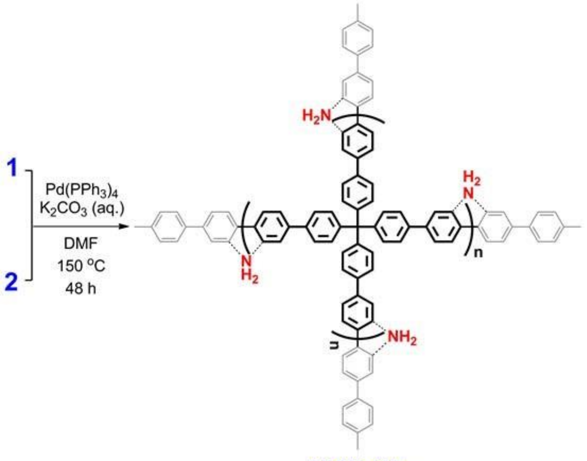

To an oven-dried 100-mL Schlenk tube were sequentially added **1** (100 mg, 0.20 mmol), **2** (132 mg, 0.40 mmol, 2 equiv.), 6.2 mL DMF and 0.8 mL aqueous K_2CO_3 solution (2.0 M). The mixture was degassed by three freeze–pump–thaw cycles. Then $Pd(PPh_3)_4$ (23 mg, 0.02 mmol, 0.1 equiv.) was added to the system quickly under argon atmosphere and the resultant reaction mixture was stirred and refluxed for 48 h at 150 °C. After cooling to room temperature, the mixture was filtrated under reduced pressure and the obtained solid was washed with H_2O (4 × 20 mL), THF (4 × 20 mL) and CH_2Cl_2 (4 × 20 mL), dried in vacuo at 100 °C, yielding the desired **PAF70-NH$_2$** (130 mg, 99% yield).

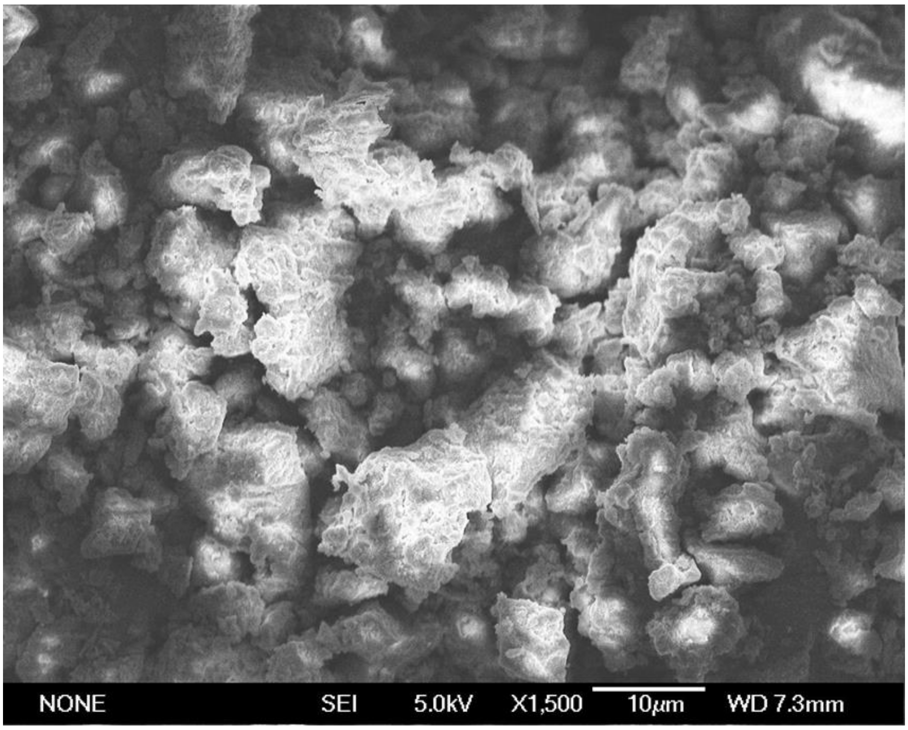

Fig. S1 SEM image of **PAF70-NH$_2$**.

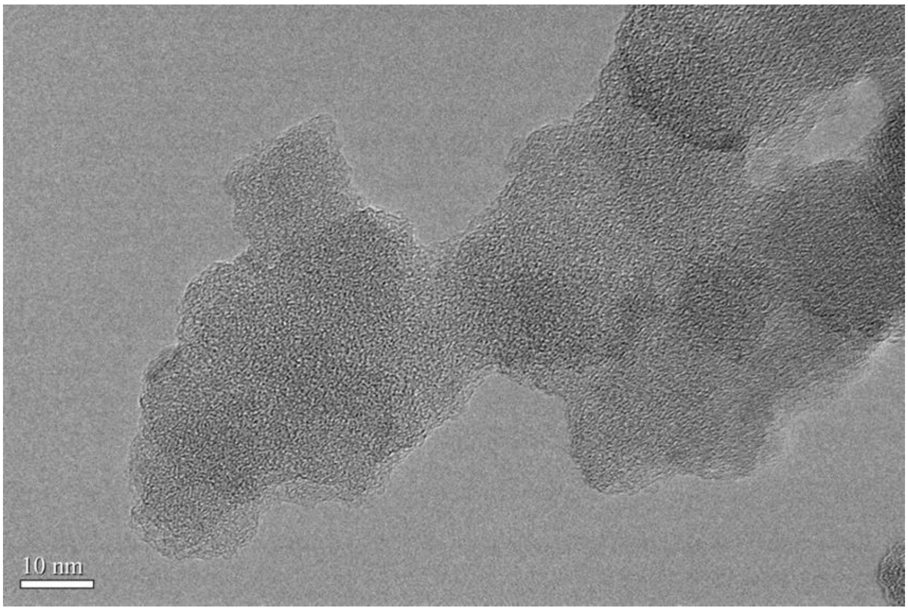

Fig. S2 TEM image of PAF70-NH$_2$.

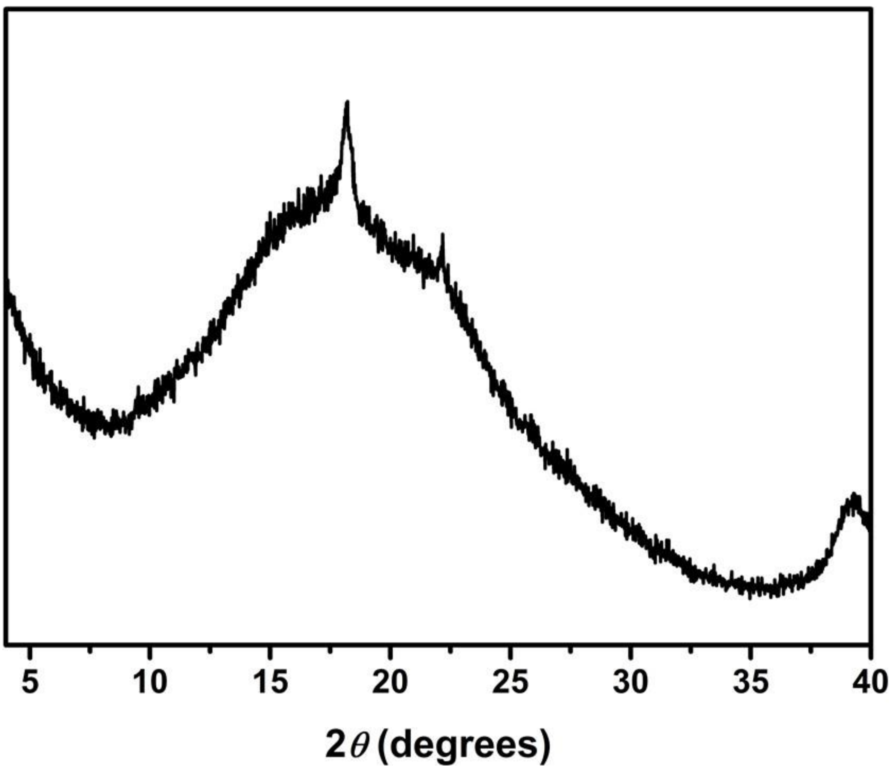

Fig. S3 The PXRD pattern of **PAF70-NH$_2$**.

3. Synthesis of PAF70-N=CPy

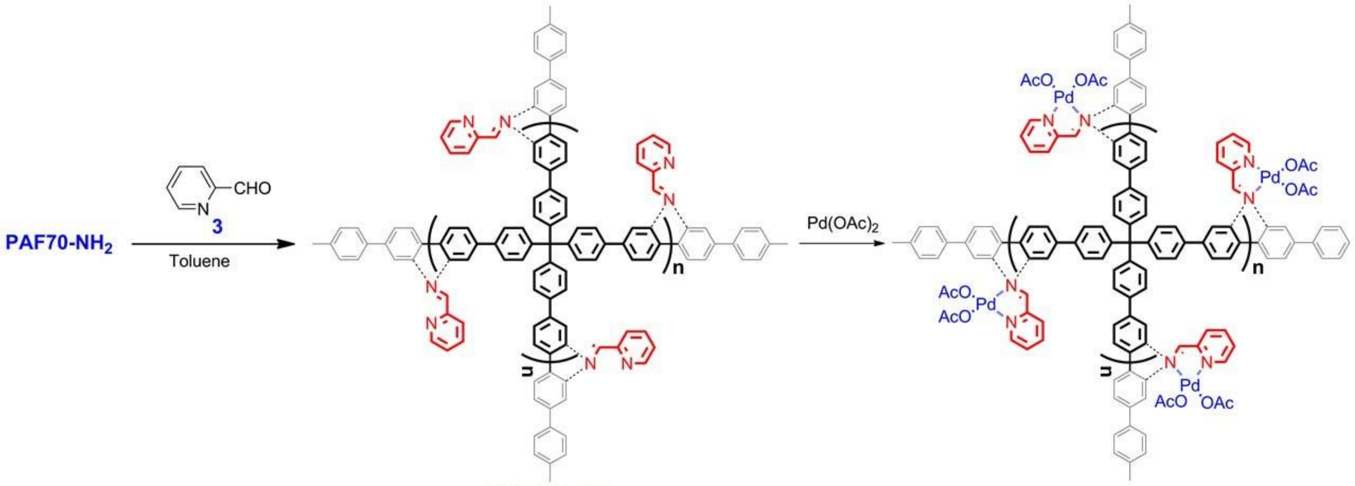

To **PAF70-NH$_2$** (200 mg) obtained above was added anhydrous toluene (5 mL) and pyridine-2-carboxaldehyde (176 uL， 1.84 mmol). The resulting mixture was stirred for 4 days at room temperature. Then the reaction mixture was centrifuged. The resultant solid was washed with CH_2Cl_2 (6 × 7 mL), dried in vacuo for 18 h at 150 °C, yielding the desired **PAF70-N=CPy** (198 mg, 66% yield).

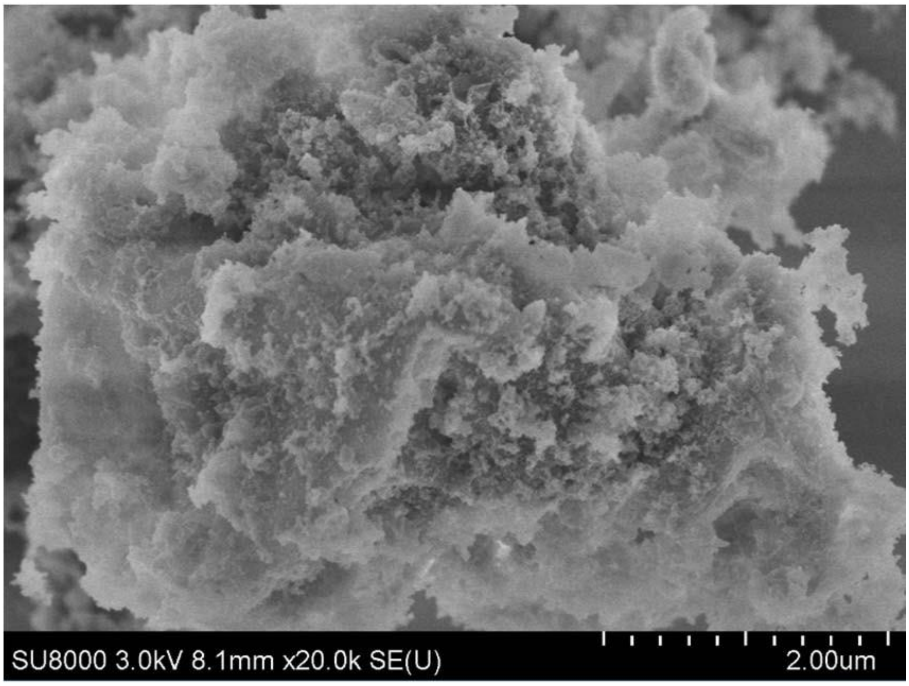

Fig. S4 SEM image of **PAF70-N=CPy**

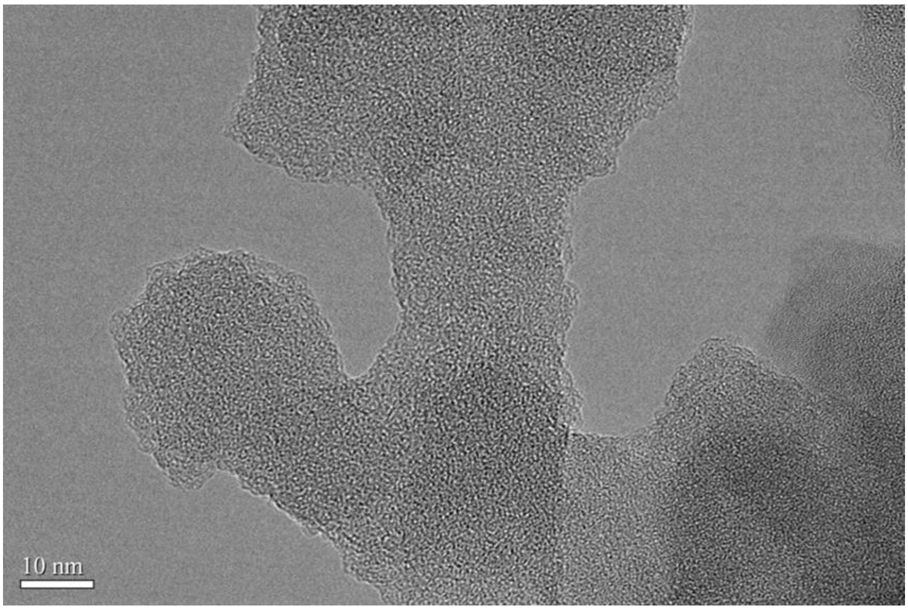

Fig. S5 TEM image of **PAF70-N=CPy**

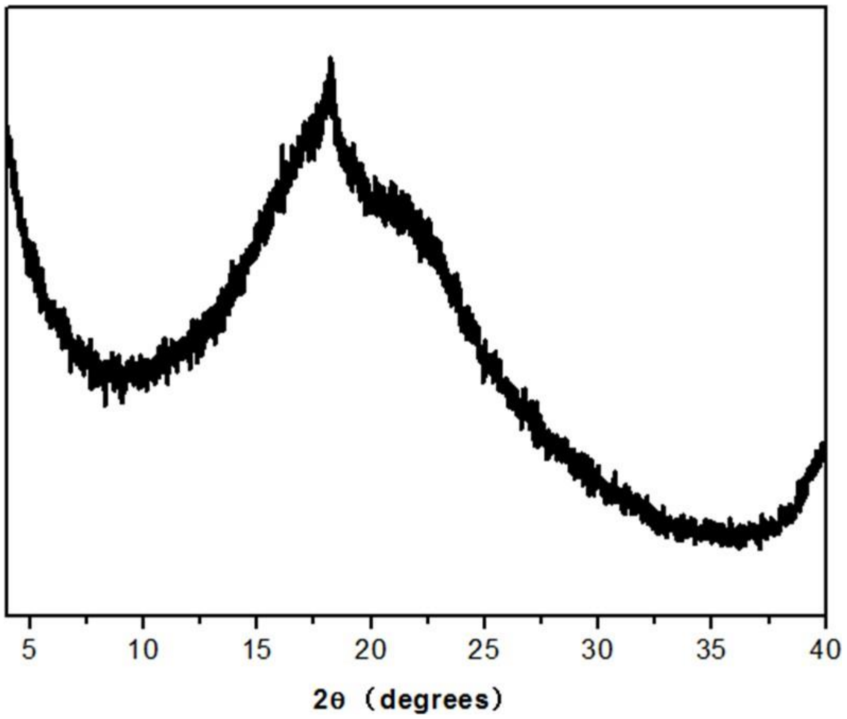

Fig. S6 The PXRD pattern of **PAF70-N=CPy**

4. Synthesis of PAF70-Pd

To **PAF70-N=CPy** (100 mg) obtained above was added a solution of palladium acetate (89 mg, 0.40 mmol) in anhydrous dichloromethane (5 mL) and the resulting mixture was stirred for 4 days at room temperature. Then the reaction mixture was centrifuged and the resultant solid was washed with CH_2Cl_2 in a soxhlet extractor for 24 h. At last, the product dried in vacuo for 18 h at 150 °C, yielding the desired **PAF70-Pd** (118 mg, 82% yield).

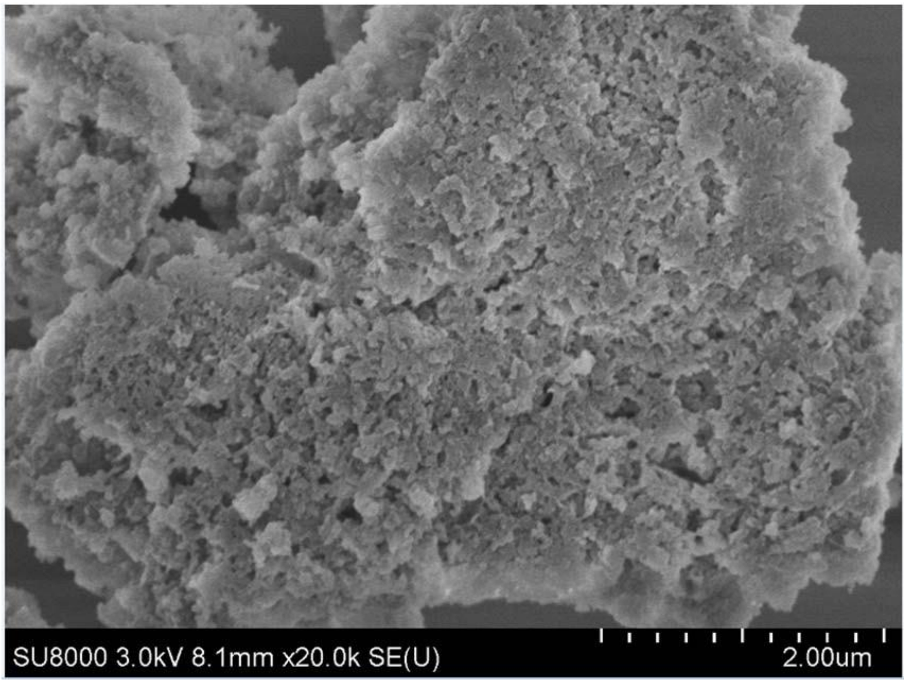

Fig. S7 SEM image of **PAF70-Pd**

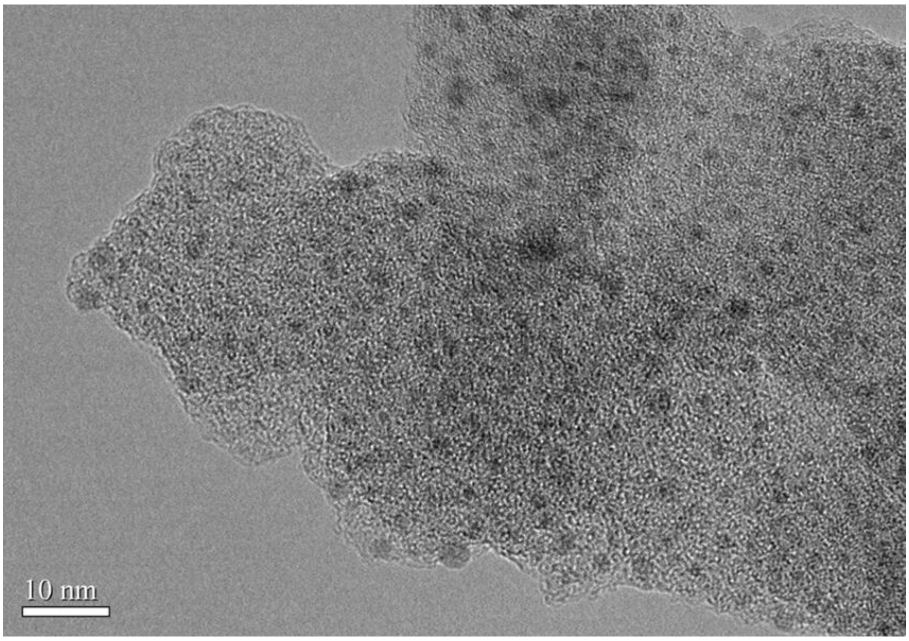

Fig. S8 TEM image of **PAF70-Pd**

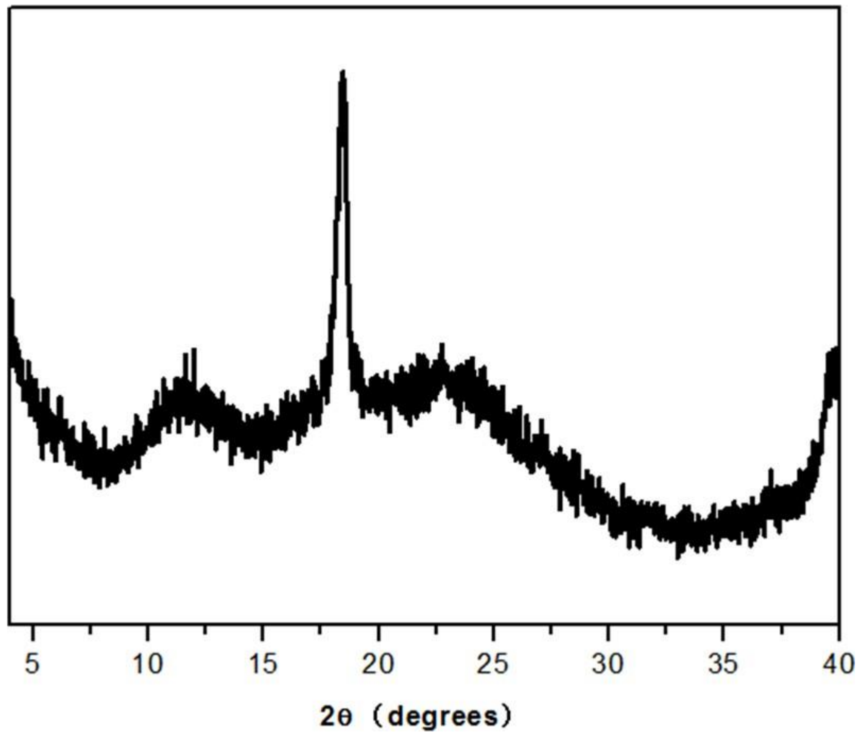

Fig. S9 The PXRD pattern of **PAF70-Pd**

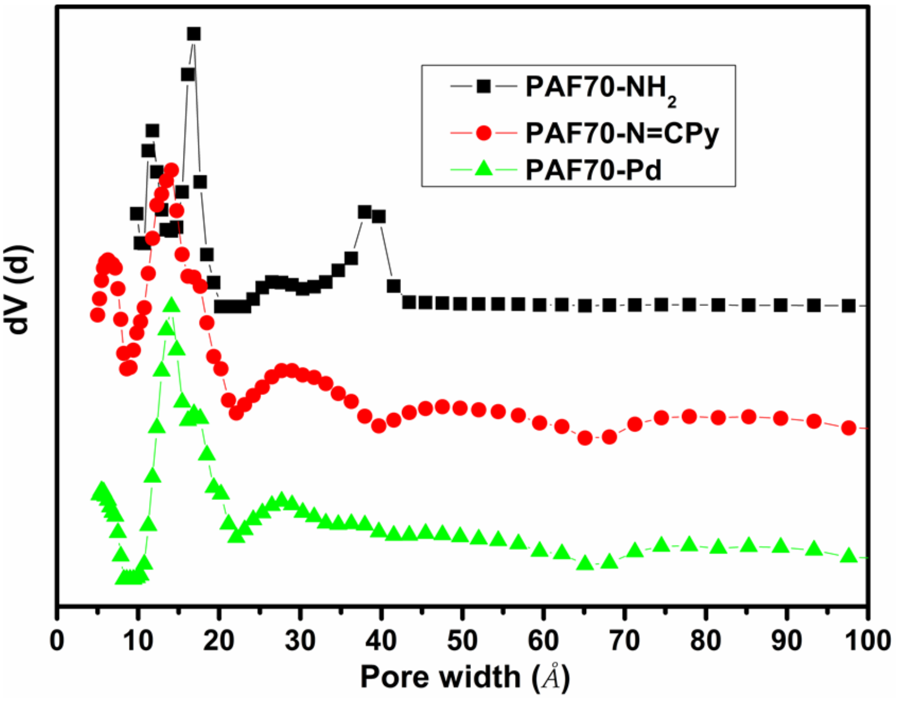

Fig. S10 Pore size distributions calculated from NLDFT of **PAF70-NH$_2$**, **PAF70-N=CPy** and **PAF70-Pd**.

5. Synthesis of PAF1-Pd

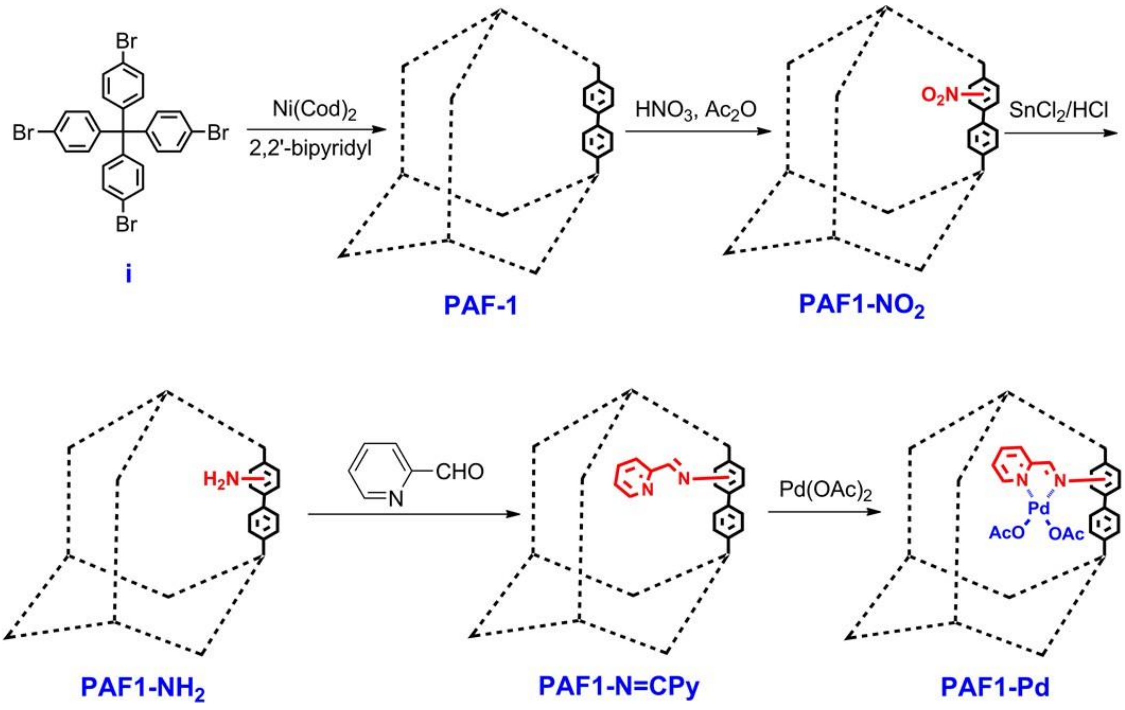

Synthesis of PAF1-NH$_2$: PAF1-NH$_2$ was synthesized according to the procedures reported in the literature with some minor modification:

Tetrakis(4-bromophenyl)methane (509 mg, 0.8 mmol) was added to a solution of 2,2'-bipyridyl (565 mg, 3.65 mmol), bis(1,5-cyclooctadiene)nickel(0) (1.0 g, 3.65 mmol), and 1,5-cyclooctadiene (0.45 mL, 3.65 mmol) in anhydrous DMF/THF (60 mL/90 mL), and the mixture was stirred for 24 h at room temperature under nitrogen atmosphere. Then 6 M HCl (60 mL) was added slowly, and the resulting mixture was stirred for 12 h. The

precipitate was collected by filtration, then washed with methanol and water, and dried in vacuo at 150 °C for 12 h to produce **PAF-1** (236 mg, 93% yield).

To a suspension of **PAF-1** (120 mg) in Ac_2O (30 mL) at an ice bath, 2.6 mL HNO_3 was gradually added. The resultant reaction mixture was then stirred at room temperature for 4 days. Subsequently, the mixture was poured into a large amount of water, and the solid was filtrated, washed with water substantially, and then dried in vacuo at 150 °C for 12 h to give **PAF-1-NO$_2$** (138 mg).

150 mg **PAF-1-NO$_2$** and 4.9 g $SnCl_2 \cdot 2H_2O$ were suspended in 30 mL ethanol. The resultant mixture was heated at 70 °C for 8 h. The solid was centrifuged and suspended in 20 mL concentrated hydrochloric acid. Then the mixture was centrifuged and the obtained solid was washed with H_2O (3 × 20 mL) and EtOH (3 × 20 mL). The product was dried in vacuo at 120 °C for 12 h to produce **PAF-1-NH$_2$** (103 mg).

Synthesis of PAF1-N=CPy: To **PAF1-NH$_2$** (60 mg) obtained above was added anhydrous toluene (2 mL) and pyridine-2-carboxaldehyde (36 uL). Then the resulting mixture was stirred for 4 days at room temperature. Then the reaction mixture was centrifuged. The resultant solid was washed with CH_2Cl_2 (5 × 7 mL) and EtOAc (5 × 7 mL), dried in vacuo at 150 °C for 12 h, yielding the desired **PAF1-N=CPy** (80 mg).

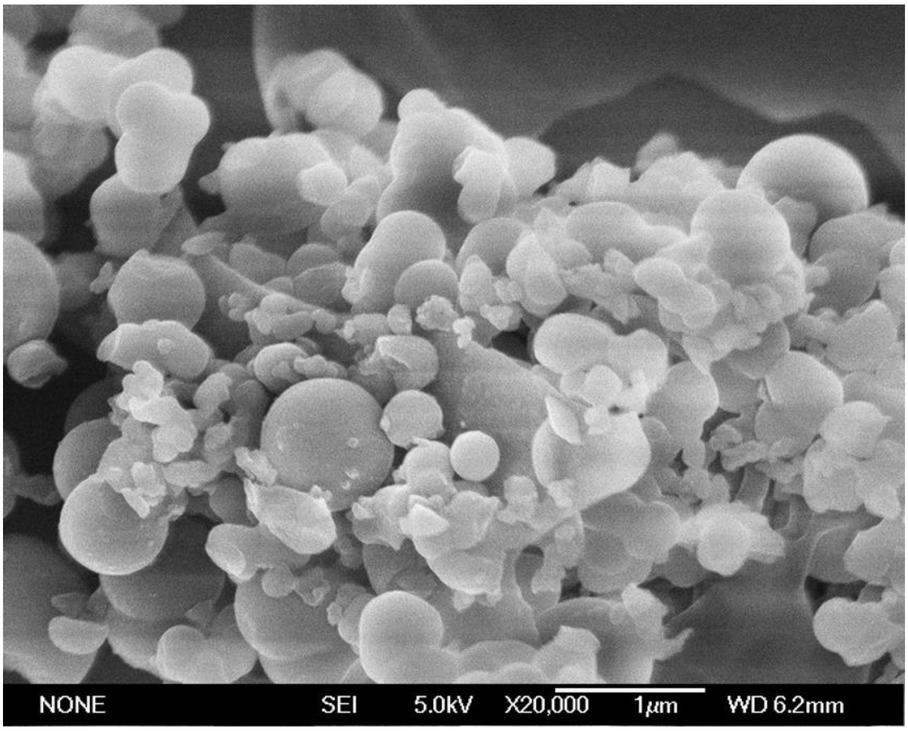

Fig. S11 SEM image of **PAF1-N=CPy**

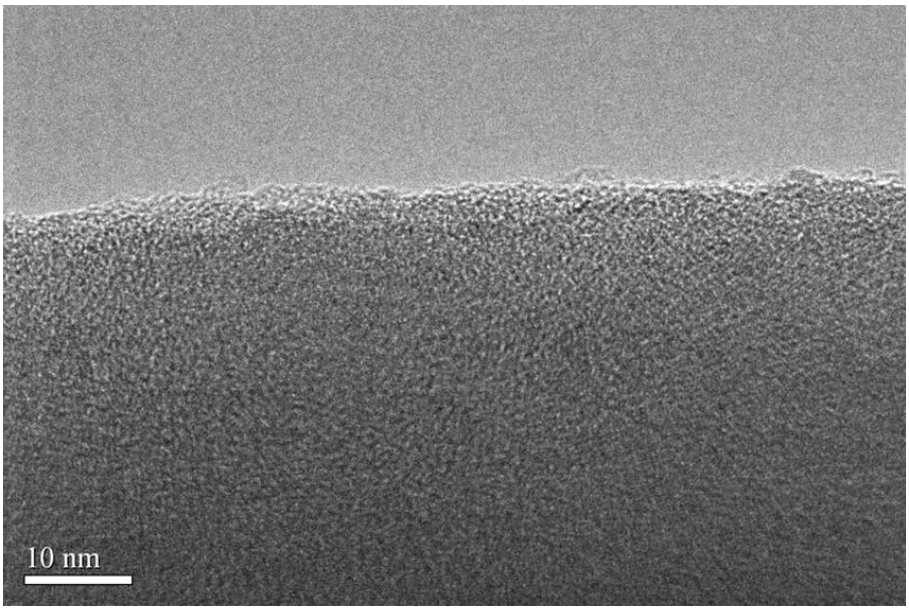

Fig. S12 TEM image of **PAF1-N=CPy**

Synthesis of PAF1-Pd: To **PAF1-N=CPy** (50 mg) obtained above was added a solution of palladium acetate (36 mg) in anhydrous dichloromethane (1.5 mL) and the resulting mixture was stirred for 4 days at room temperature. Then the reaction mixture was centrifuged and the resultant solid was washed with CH_2Cl_2 in a soxhlet extractor for 24 h. Then, the solid was dried in vacuo at 150 °C for 12 h, yielding the desired **PAF1-Pd** (59 mg).

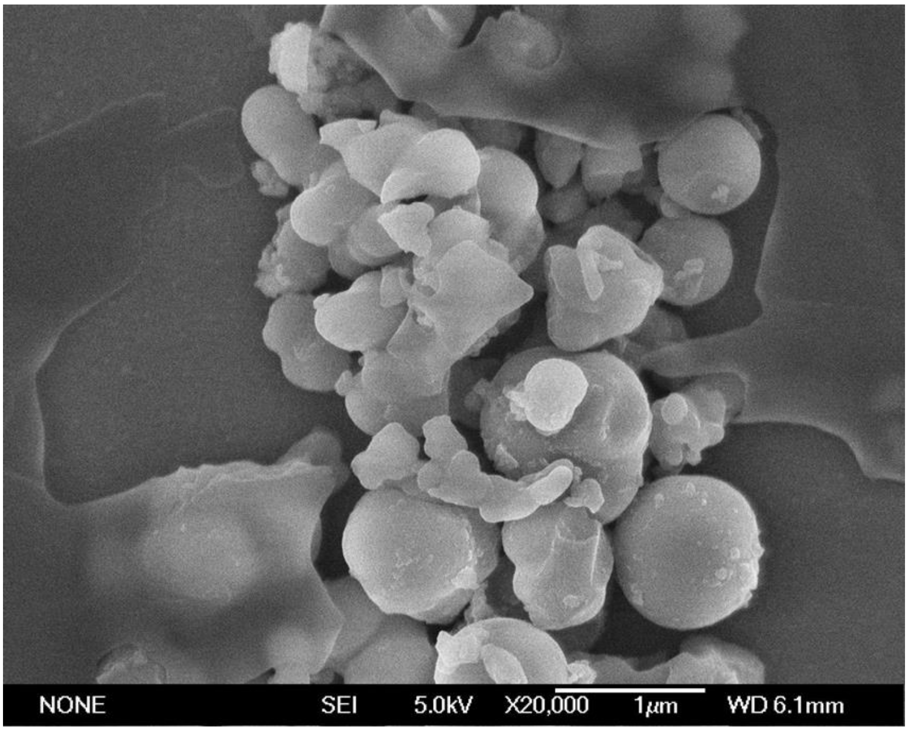

Fig. S13 SEM image of PAF1-Pd

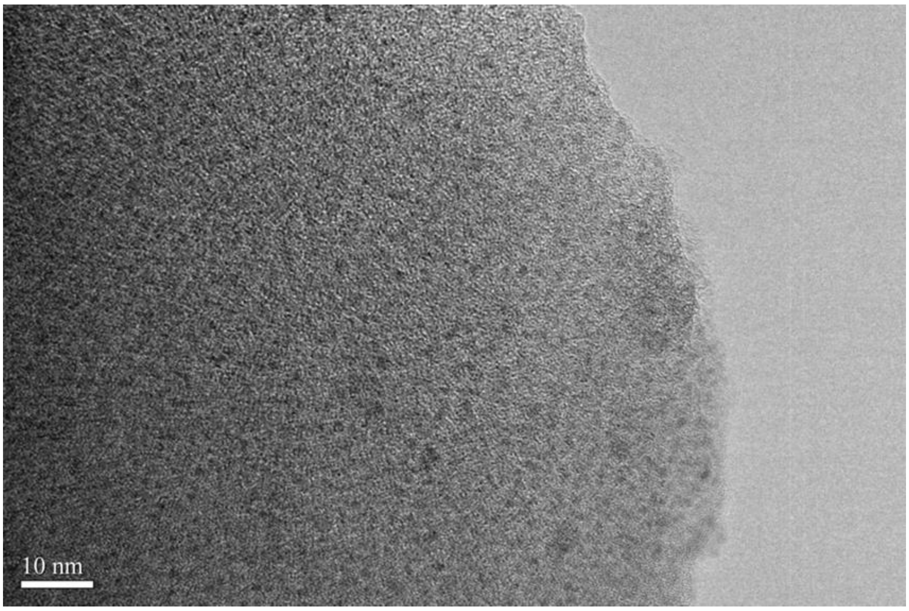

Fig. S14 TEM image of PAF1-Pd

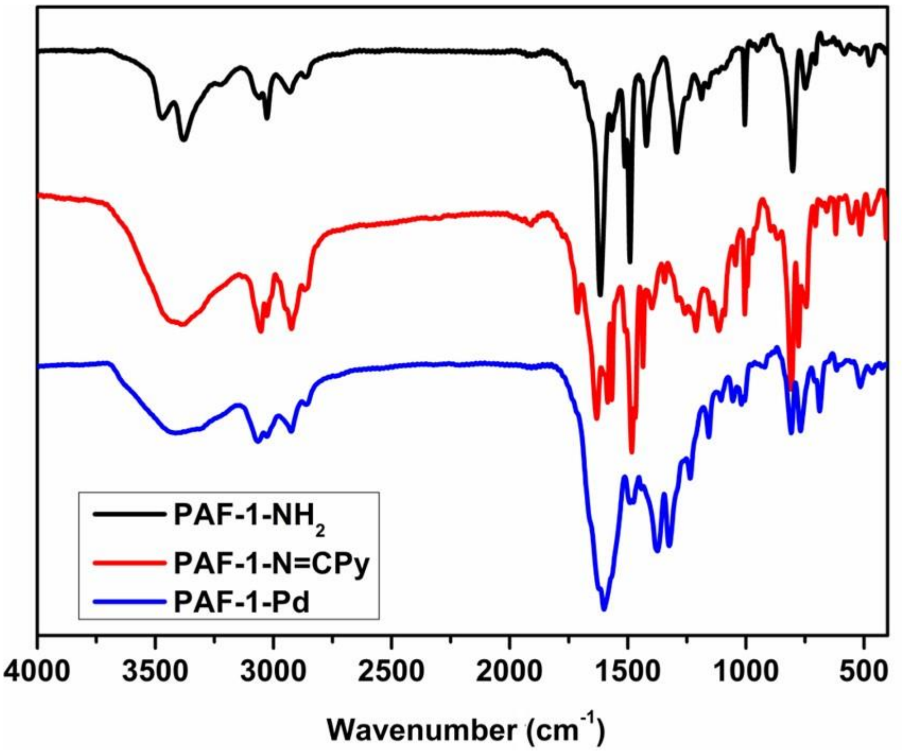

Fig. S15 FT-IR spectra of PAF1-NH_2 (black), PAF1-N=CPy (red), and PAF1-Pd (blue)

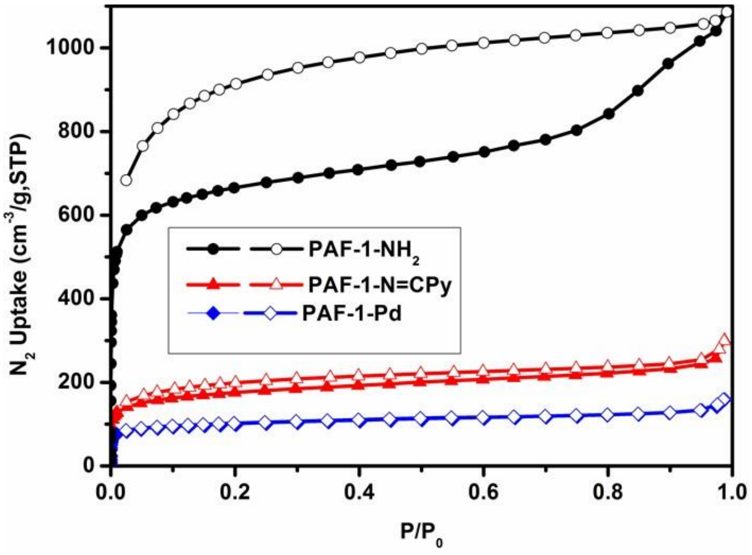

Fig. S16 Nitrogen adsorption (solid symbols)–desorption (open symbols) isotherms measured at 77 K of PAF1-NH_2 (black), PAF1-N=CPy (red) and PAF1-Pd (blue).

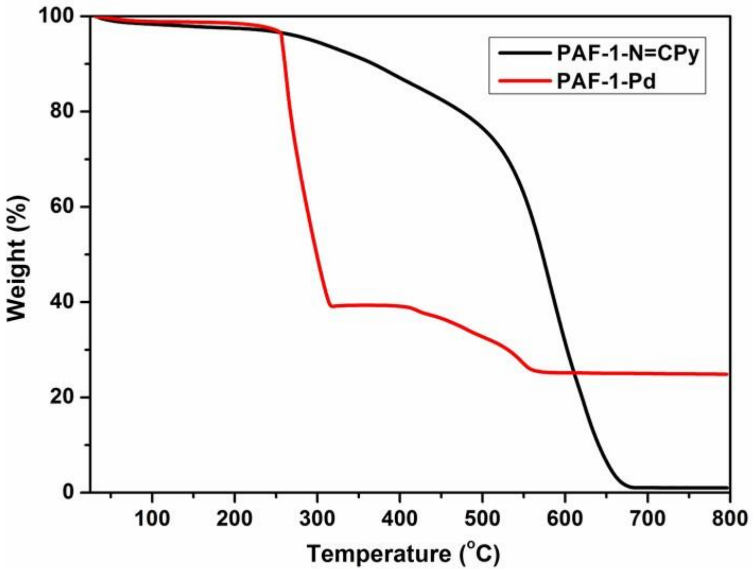

Fig. S17 TGA plots of PAF1-N=CPy and PAF1-Pd.

6. PAF70-Pd Catalyzed Suzuki-Miyaura Coupling Reaction

■ General experimental procedure was described as follows:

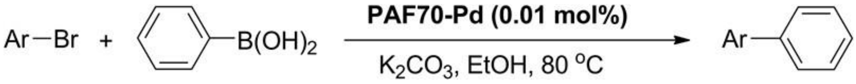

To an oven-dried 10 mL reaction flask were sequentially added aryl bromide **1–11a** (2.6 mmol, 1 equiv.), phenylboronic acid (476 mg, 3.9 mmol, 1.5 equiv.), potassium carbonate (719 mg, 5.2 mmol, 2 equiv) , the catalyst **PAF70-Pd** (0.12 mg, 0.00026 mmol Pd unit, 0.0001 equiv) and EtOH (10.0 mL) were added to the reaction system.

The reaction mixture was stirred at 80 °C for indicated time. Then cooled to room temperature. The product was collected by centrifugation, the solid was washed with CH_2Cl_2 (5 × 7 mL). The combined solution was concentrated under reduced pressure. the crude product was purified by flash chromatography over silica gel to

give the product **1–11b**.

1b: ^{1}H NMR (300 MHz, $CDCl_3$): δ = 8.31 (d, J = 9.0 Hz, 2H), 7.74 (d, J = 9.0 Hz, 2H), 7.65–7.60 (m, 2H), 7.54–7.41 ppm (m, 3H);

2b: ^{1}H NMR (300 MHz, $CDCl_3$): δ = 10.06 (s, 1H), 7.96 (d, J=8.4 Hz, 2H), 7.76 (d, J=8.4 Hz, 2H), 7.67–7.62 (m, 2H), 7.52–7.39 ppm (m, 3H);

3b: ^{1}H NMR (300 MHz, $CDCl_3$): δ = 7.69 (s, 4H), 7.67–7.63 (m, 4H), 7.50–7.43 (m, 4H), 7.40–7.33 ppm (m, 2H);

4b: ^{1}H NMR (300 MHz, $CDCl_3$): δ = 7.63–7.51 (m, 4H), 7.48–7.40 (m, 2H), 7.39–7.31 (m, 1H), 7.18–7.08 ppm (m, 2H);

5b: ^{1}H NMR (300 MHz, $CDCl_3$): δ = 7.76–7.66 (m, 4H), 7.62–7.56 (m, 2H), 7.52–7.39 ppm (m, 3H);

6b: ^{1}H NMR (300 MHz, $CDCl_3$): δ = 7.59–7.51 (m, 4H), 7.46–7.39 (m, 2H), 7.35–7.28 (m, 1H), 7.02–6.96 (m, 2H), 3.86 ppm (s, 3H);

7b: ^{1}H NMR (300 MHz, $CDCl_3$): δ = 7.60–7.54 (m, 2H), 7.52–7.46 (m, 2H), 7.46–7.38 (m, 2H), 7.36–7.28 (m, 1H), 7.28–7.21 (m, 2H), 2.40 ppm (s, 3H);

8b: ^{1}H NMR (300 MHz, $CDCl_3$): 7.62–7.55 (m, 4H), 7.49–7.40 (m, 4H), 7.39–7.30 (m, 1H), 4.96 (q, J = 6.6 Hz, 1H), 1.75 (brs, 1H), 1.55 ppm (d, J = 6.6 Hz, 3H);

9b: ^{1}H NMR (300 MHz, $CDCl_3$): δ = 7.67–7.60 (m, 4H), 7.52–7.43 (m, 4H), 7.42–7.34 ppm (m, 2H);

7. Recyclability of PAF70-Pd for Catalyzing Suzuki-Miyaura Coupling Reaction

Table S1 The recycle test of **PAF70-Pd** as catalyst in the Suzuki-Miyaura coupling reaction [a]

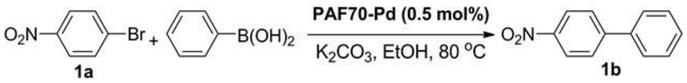

Cycle	Time	Yield[b]
1	7min	95%
2	7min	96%
3	8min	95%

[a]Reaction condition: For cycles 1-3, a solution of 2.5 mmol **1a**, 3.75 mmol phenylboronic acid, 5.0 mmol K_2CO_3, and the catalysts (0.5 mol%) in 10 mL of EtOH was stirred at 80 °C for indicated time. [b]The isolated yield.

■ General experimental procedure was described as follows:

The experimental procedure for cycle 1: To an oven-dried 25 mL reaction flask were sequentially added 1-Bromo-4-nitrobenzene (525 mg, 2.6 mmol, 1 equiv.), phenylboronic acid (476 mg, 3.9 mmol, 1.5 equiv.), potassium carbonate (719 mg, 5.2 mmol, 2 equiv.), the catalyst **PAF70-Pd** (6.0 mg, 0.013 mmol Pd unit, 0.005 equiv) and EtOH (10.0 mL) were added to the reaction system. The reaction mixture was stirred at 80 °C for 7 min and then cooled to room temperature. After centrifugation, the solid was washed with CH_2Cl_2 (5 × 7 mL). The combined organic solution was concentrated under reduced pressure and the crude product could be further purified by flash chromatography over silica gel to give the pure product **1b**.

The recovery procedure of catalyst: The solid obtained from centrifugation above was washed with H_2O (2 × 7 mL), and EtOH (2 × 7 mL), providing the recovered catalyst **PAF70-Pd**.

The cycles 2-3: The above-mentioned recovered catalyst **PAF70-Pd** was reused into the same reaction and the dosages of all the reactants were the same as that in cycle 1. The reaction mixture was stirred at 80 °C for indicated time.

8. Comparison with the previously reported POPs immobilized Pd catalysts

Table S2 Pd contents and the catalytic performances for catalyzing Suzuki-Miyaura coupling reaction of **PAF70-Pd**, **PAF1-Pd** and other published POPs immobilized Pd catalysts[a]

catalyst	Pd loading	TOF (h^{-1})[b]	TOF (h^{-1})[c]	Size selectivity
PAF70-Pd	23.1 wt%	23280	28800	Yes
PAF1-Pd	25.1 wt%	--	--	--
Pd@CNPCs[1]	--	--	327	--
Pd/COF-LZU1[2]	7.13 wt %	65	65	--
Pd-CIN-1[3]	0.60 wt %	2878	980	--
MsMOP-1[4]	5.01 wt %	351	319	--
Pd/MPP[5]	4.56 wt%	960	960	--
KAPs(Ph-PPh3)-Pd[6]	0.7wt%	--	--	--
PdII/UOF-1[7]	16.87 wt %	61	--	--
PdII/UOF-2[7]	16.83 wt %	62	--	--
Pd(0)/MCoS-1[8]	1.0 wt%	86	102	--
ZrDMTD-Pd[9]	8.7 wt%	25[d]	--	--
Pd/H_2P-Bph-COF[10]	12.87 wt %	129	--	--
Pd@N=P[11]	1.0 wt%	--	<=1054?	Yes
Pd@N=P=O[11]	0.89 wt%	--	<=1187?	--
PPI-1-NPy-Pd[12]	1.76 wt %	--	450	--
PPI-2-NPy-Pd[12]	3.42 wt%	--	580	--
Pd(II)@bbp-MOP[13]	5.89 wt%	1960	1452	--
Pd/SNW1[14]	1.23 wt%	--	--	--
Pd(OAc)$_2$@COF-300[15]	1.74 wt%	--	3000	--
Pd/POP[16]	3 wt%,	--	219	--
Bpy-Pd-POP-1[17]	15.05 wt%	78	248	--
Bpy-Pd-POP-2[17]	16.29 wt%	--	246	
Pd@NHC-MOP[18]	13.77 wt %	59	248	--
Pd/Cy-pip[19]	--	100	88	--
Pd/CM[20]	5 wt%	31	--	--
Pd/AM[20]	3.9 wt%	8	--	--

[a]The TOF was calculated by the Suzuki-Miyaura coupling reaction under their respective reaction condition. [b]The reactants were 4-bromonitrobenzene and phenylboronic acid. [c]The reactants were bromobenzene and phenylboronic acid.

9. References

1. P. Zhang, Z. Weng, J. Guo and C. Wang, *Chem. Mater.*, 2011, **23**, 5243-5249.
2. S. Y. Ding, J. Gao, Q. Wang, Y. Zhang, W. G. Song, C. Y. Su and W. Wang, *J. Am. Chem. Soc.*, 2011, **133**, 19816-19822.
3. M. K. Bhunia, S. K. Das, P. Pachfule, R. Banerjee and A. Bhaumik, *Dalton Trans.*, 2012, **41**, 1304-1311.
4. H. Li, B. Xu, X. Liu, S. A, C. He, H. Xia and Y. Mu, *J. Mater. Chem. A*, 2013, **1**, 14108.
5. Q. Song, Y. Jia, B. Luo, H. He and L. Zhi, *Small*, 2013, **9**, 2460-2465.
6. Z. Guan, B. Li, G. Hai, X. Yang, T. Li and B. Tan, *RSC Adv.*, 2014, **4**, 36437.
7. L. Li, Z. Chen, H. Zhong and R. Wang, *Chem. Eur. J.*, 2014, **20**, 3050-3060.
8. A. S. Roy, J. Mondal, B. Banerjee, P. Mondal, A. Bhaumik and S. M. Islam, *Appl. Catal., A*, 2014, **469**, 320-327.
9. B. Gui, K. K. Yee, Y. L. Wong, S. M. Yiu, M. Zeller, C. Wang and Z. Xu, *Chem. Commun.*, 2015, **51**, 6917-6920.
10. Y. Hou, X. Zhang, J. Sun, S. Lin, D. Qi, R. Hong, D. Li, X. Xiao and J. Jiang, *Microporous Mesoporous Mater.*, 2015, **214**, 108-114.
11. X. Jiang, W. Zhao, W. Wang, F. Zhang, X. Zhuang, S. Han and X. Feng, *Polym. Chem.*, 2015, **6**, 6351-6357.
12. E. Rangel Rangel, E. M. Maya, F. Sánchez, J. G. de la Campa and M. Iglesias, *Green Chem.*, 2015, **17**, 466-473.
13. Q. Wen, T. Y. Zhou, Q. L. Zhao, J. Fu, Z. Ma and X. Zhao, *Macromol. Rapid Commun.*, 2015, **36**, 413-418.
14. M. Shunmughanathan, P. Puthiaraj and K. Pitchumani, *ChemCatChem*, 2015, **7**, 666-673.
15. R. S. B. Gonçalves, A. B. V. de Oliveira, H. C. Sindra, B. S. Archanjo, M. E. Mendoza, L. S. A. Carneiro, C. D. Buarque and P. M. Esteves, *ChemCatChem*, 2016, **8**, 743-750.
16. X. Ren, S. Kong, Q. Shu and M. Shu, *Chin. J. Chem.*, 2016, **34**, 373-380.
17. C.-A. Wang, Y.-F. Han, Y.-W. Li, K. Nie, X.-L. Cheng and J.-P. Zhang, *RSC Adv.*, 2016, **6**, 34866-34871.
18. C.-A. Wang, Y.-W. Li, X.-M. Hou, Y.-F. Han, K. Nie and J.-P. Zhang, *ChemistrySelect*, 2016, **1**, 1371-1376.
19. Z.-L. Du, Q.-Q. Dang and X.-M. Zhang, *Ind. Eng. Chem. Res.*, 2017, **56**, 4275-4280.
20. Y. Monguchi, F. Wakayama, S. Ueda, R. Ito, H. Takada, H. Inoue, A. Nakamura, Y. Sawama and H. Sajiki, *RSC Adv.*, 2017, **7**, 1833-1840.

9. Copies of NMR Spectra

See the next page!

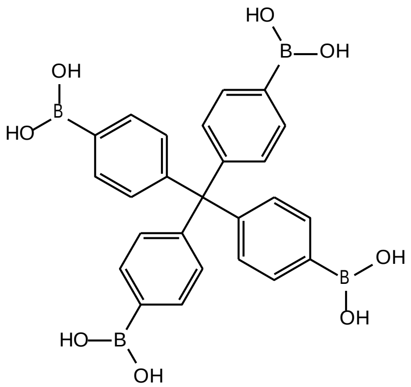
HO
B
OH
OH
B
HO
OH
B
OH
HO
B
OH

1

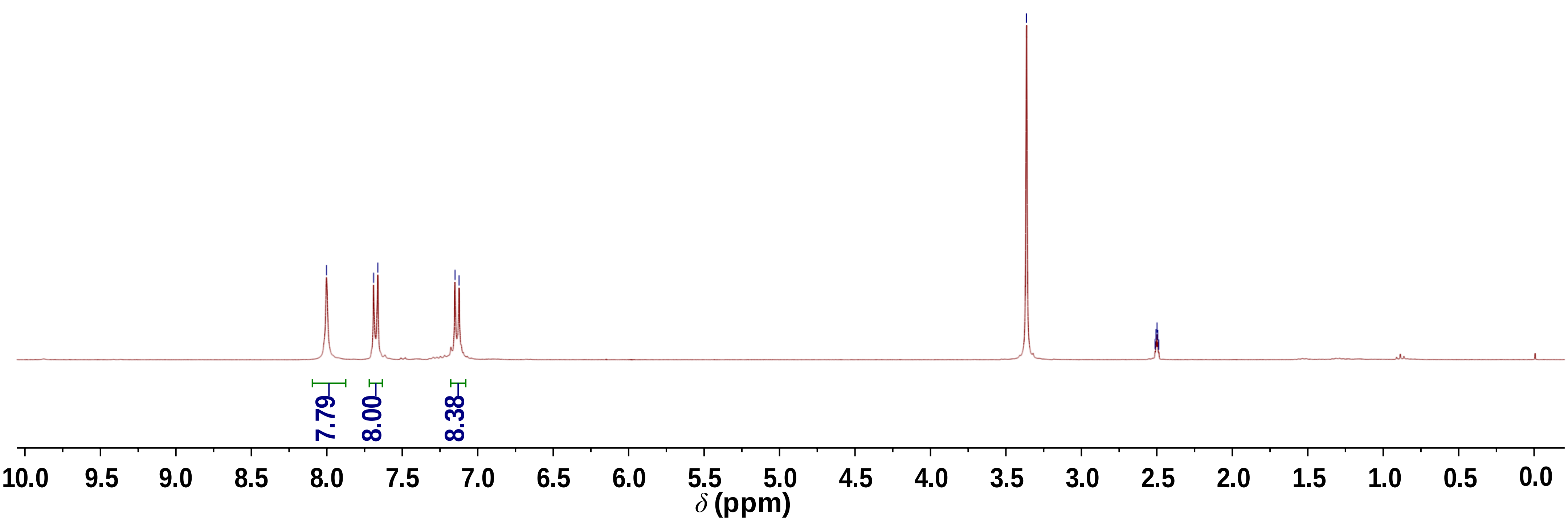
8.001
7.690
7.662
7.151
7.123
3.364
2.512
2.506
2.500
2.494
2.488
7.79
8.00
8.38
10.0
9.5
9.0
8.5
8.0
7.5
7.0
6.5
6.0
5.5
5.0
4.5
4.0
3.5
3.0
2.5
2.0
1.5
1.0
0.5
0.0
δ (ppm)

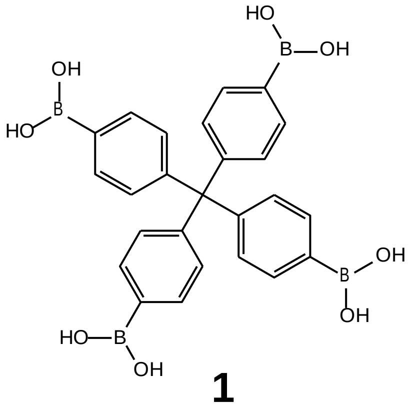

1

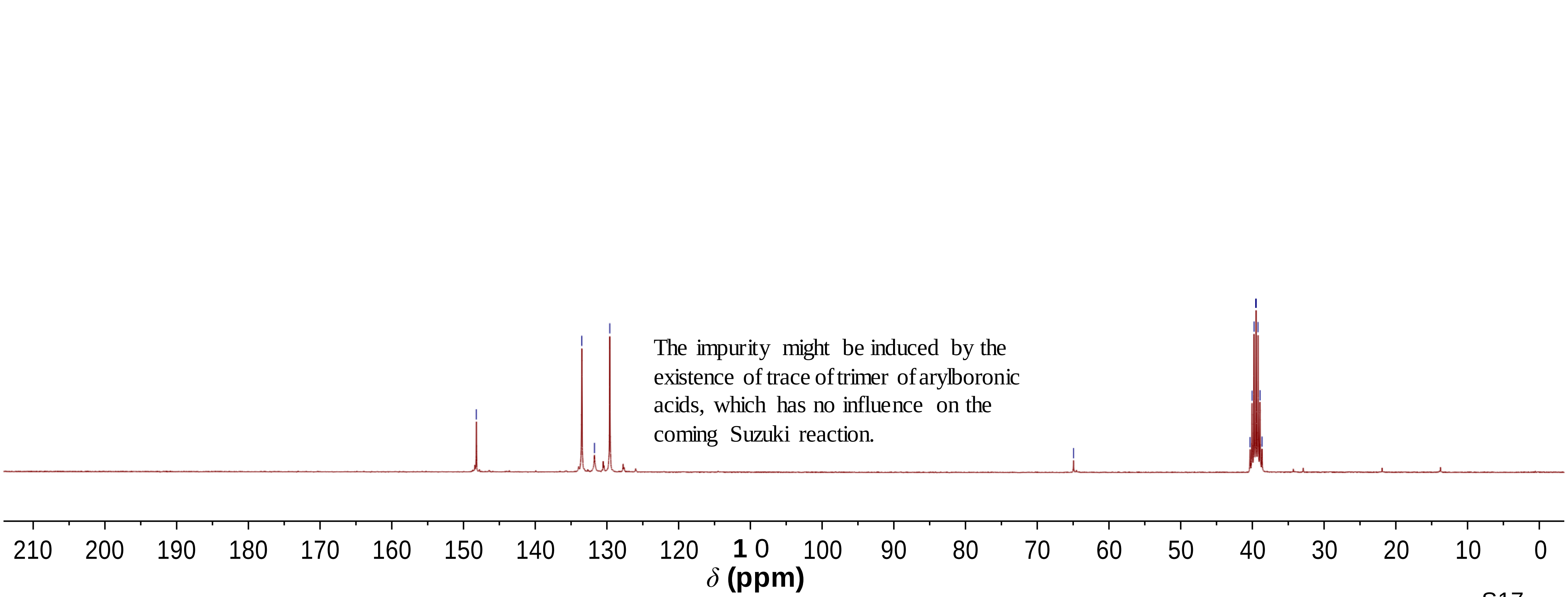
148.22
133.51
131.75
129.61
64.95
40.35
40.07
39.79
39.51
39.23
38.95
38.67
The impurity might be induced by the existence of trace of trimer of arylboronic acids, which has no influence on the coming Suzuki reaction.
210 200 190 180 170 160 150 140 130 120 1 0 100 90 80 70 60 50 40 30 20 10 0
δ (ppm)

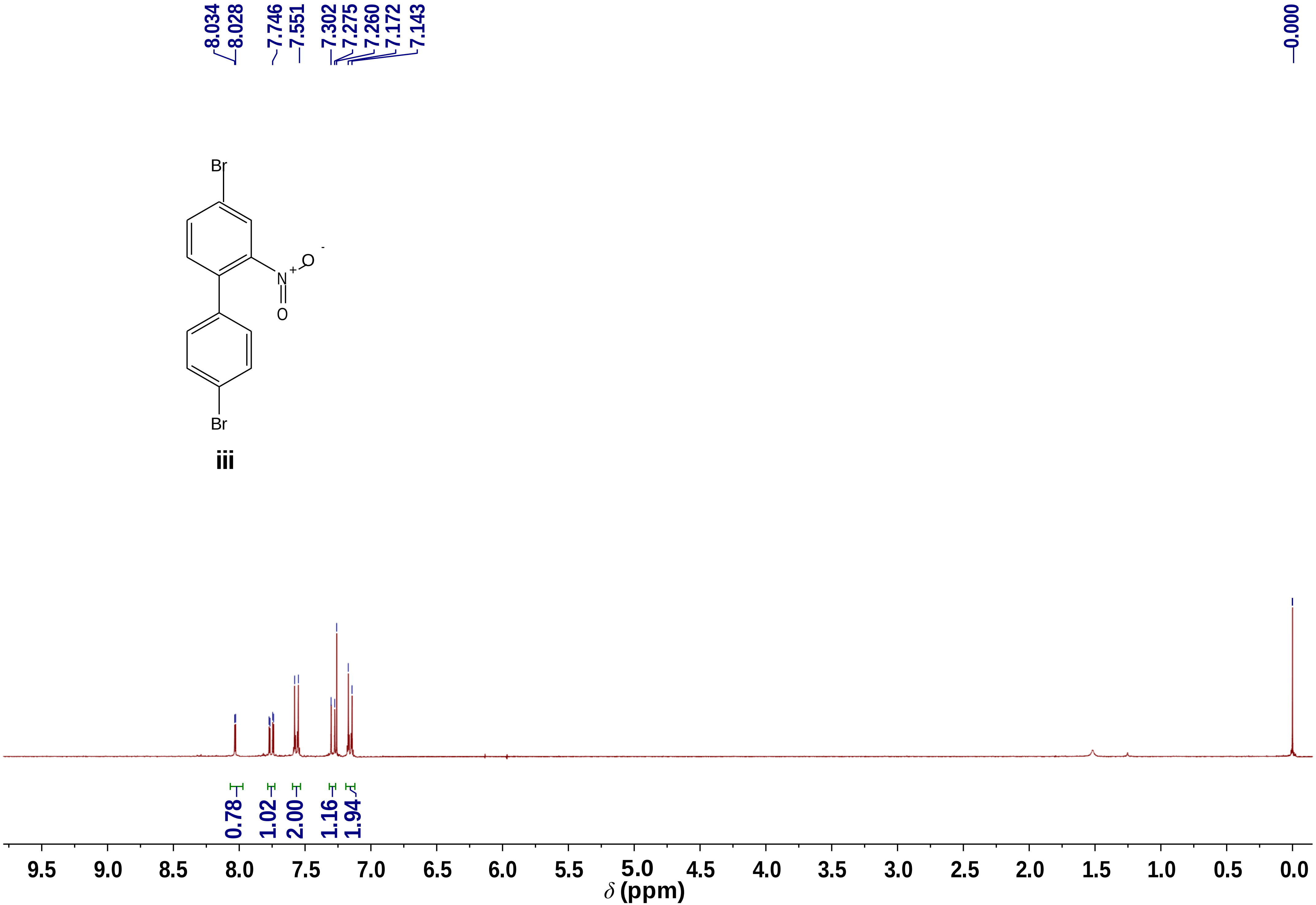
8.034
8.028
7.746
7.551
7.302
7.275
7.260
7.172
7.143
0.000
Br
N
+
O
-
O
Br
iii
0.78
1.02
2.00
1.16
1.94
9.5
9.0
8.5
8.0
7.5
7.0
6.5
6.0
5.5
5.0
4.5
4.0
3.5
3.0
2.5
2.0
1.5
1.0
0.5
0.0
δ (ppm)

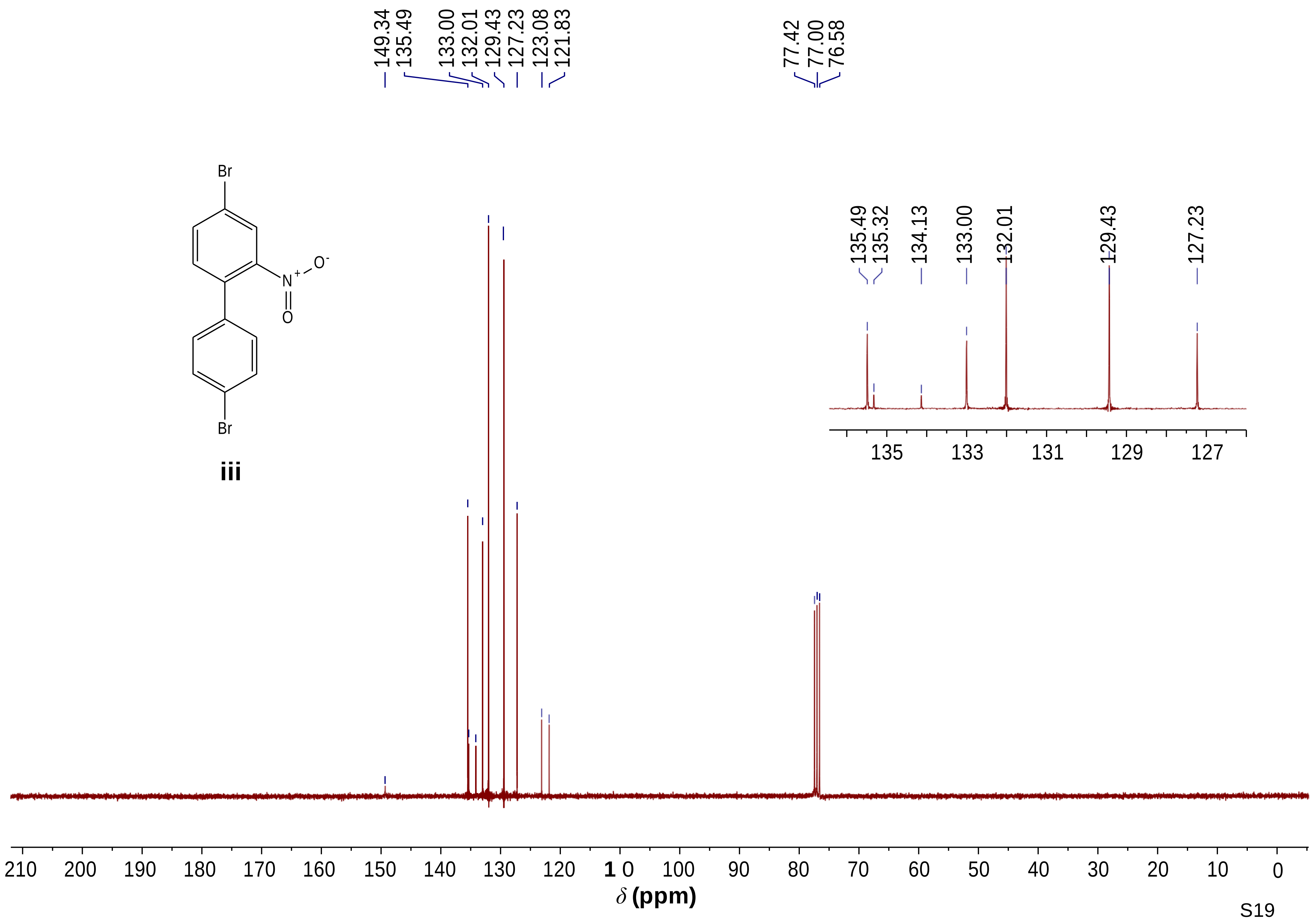
149.34
135.49
133.00
132.01
129.43
127.23
123.08
121.83
77.42
77.00
76.58
Br
N
O
O
Br
iii
135.49
135.32
134.13
133.00
132.01
129.43
127.23
135
133
131
129
127
210
200
190
180
170
160
150
140
130
120
1 0
100
90
80
70
60
50
40
30
20
10
0
δ (ppm)

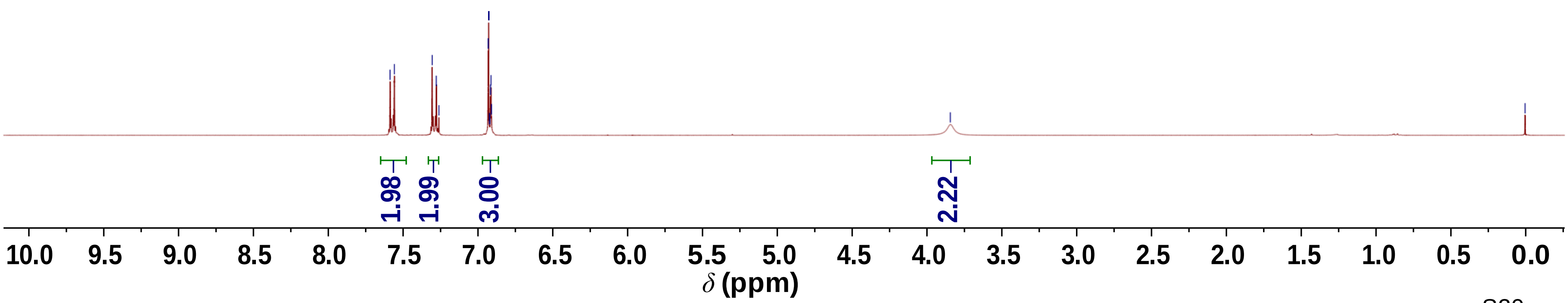
7.586
7.558
7.260
6.931
6.928
6.922
6.921
6.915
6.912
6.909
3.844
0.005
1.98
1.99
3.00
2.22
10.0 9.5 9.0 8.5 8.0 7.5 7.0 6.5 6.0 5.5 5.0 4.5 4.0 3.5 3.0 2.5 2.0 1.5 1.0 0.5 0.0
δ (ppm)

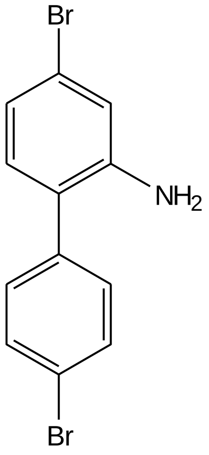
Br
NH2
Br

2

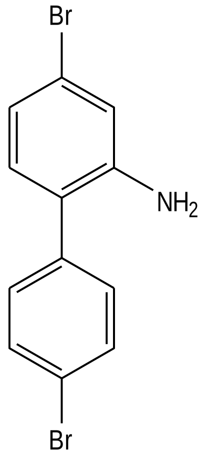
Br
NH2
Br

2

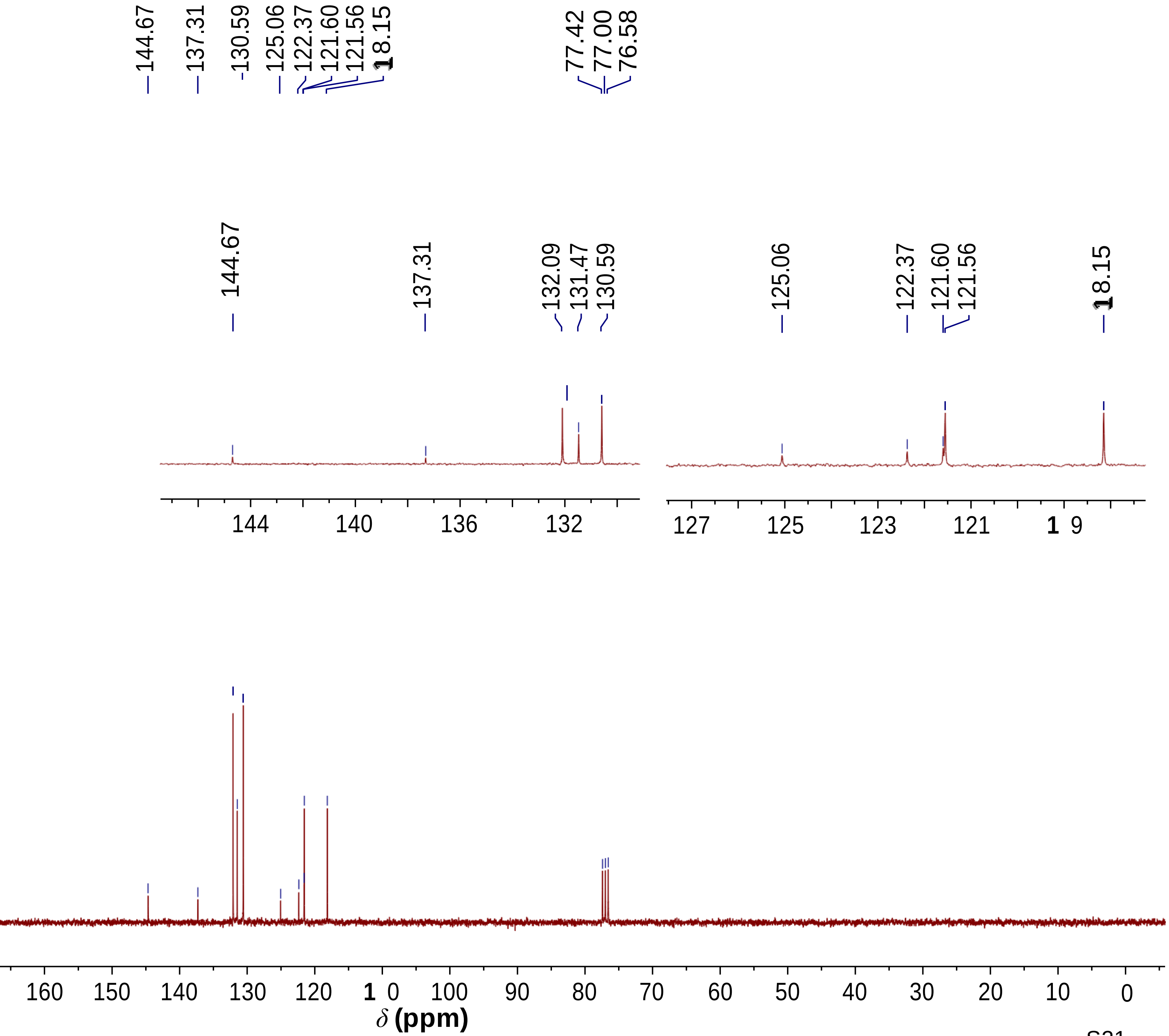
144.67
137.31
130.59
125.06
122.37
121.60
121.56
18.15
77.42
77.00
76.58
132.09
131.47
δ (ppm)

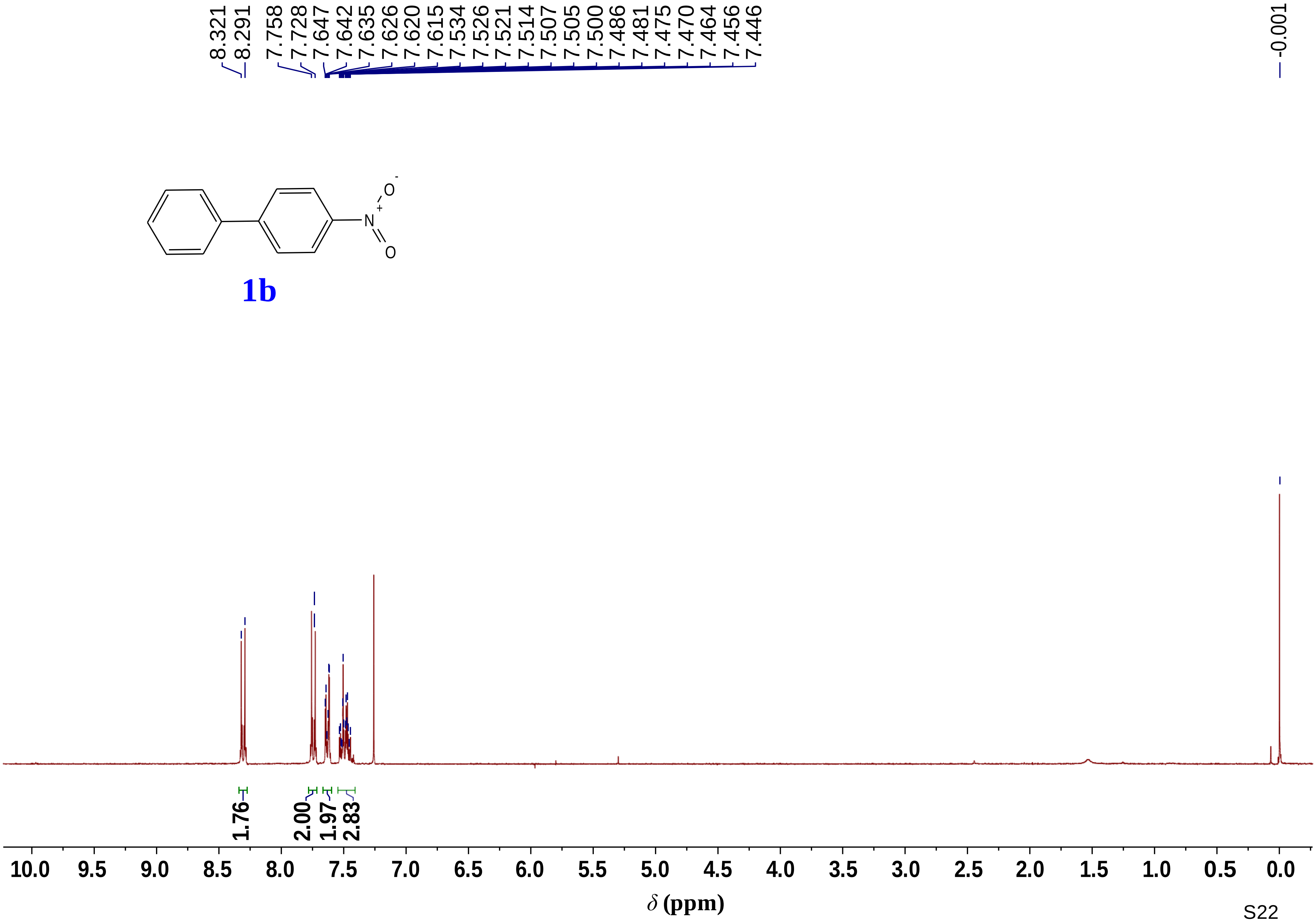
8.321
8.291
7.758
7.728
7.647
7.642
7.635
7.626
7.620
7.615
7.534
7.526
7.521
7.514
7.507
7.505
7.500
7.486
7.481
7.475
7.470
7.464
7.456
7.446
-0.001
1b
1.76
2.00
1.97
2.83
10.0 9.5 9.0 8.5 8.0 7.5 7.0 6.5 6.0 5.5 5.0 4.5 4.0 3.5 3.0 2.5 2.0 1.5 1.0 0.5 0.0
δ (ppm)

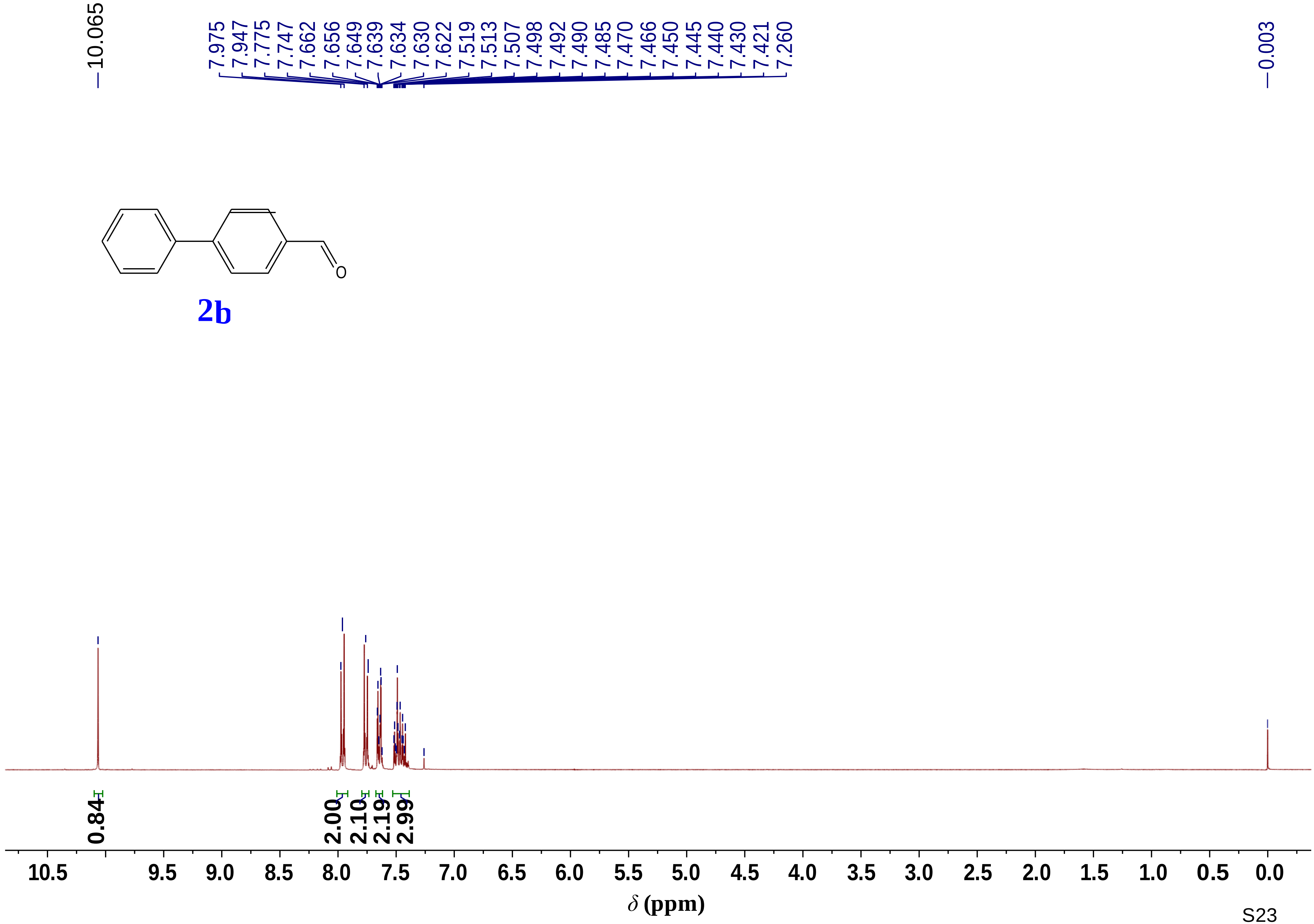
10.065
7.975
7.947
7.775
7.747
7.662
7.656
7.649
7.639
7.634
7.630
7.622
7.519
7.513
7.507
7.498
7.492
7.490
7.485
7.470
7.466
7.450
7.445
7.440
7.430
7.421
7.260
0.003
O
2b
0.84
2.00
2.10
2.19
2.99
10.5
9.5
9.0
8.5
8.0
7.5
7.0
6.5
6.0
5.5
5.0
4.5
4.0
3.5
3.0
2.5
2.0
1.5
1.0
0.5
0.0
δ (ppm)

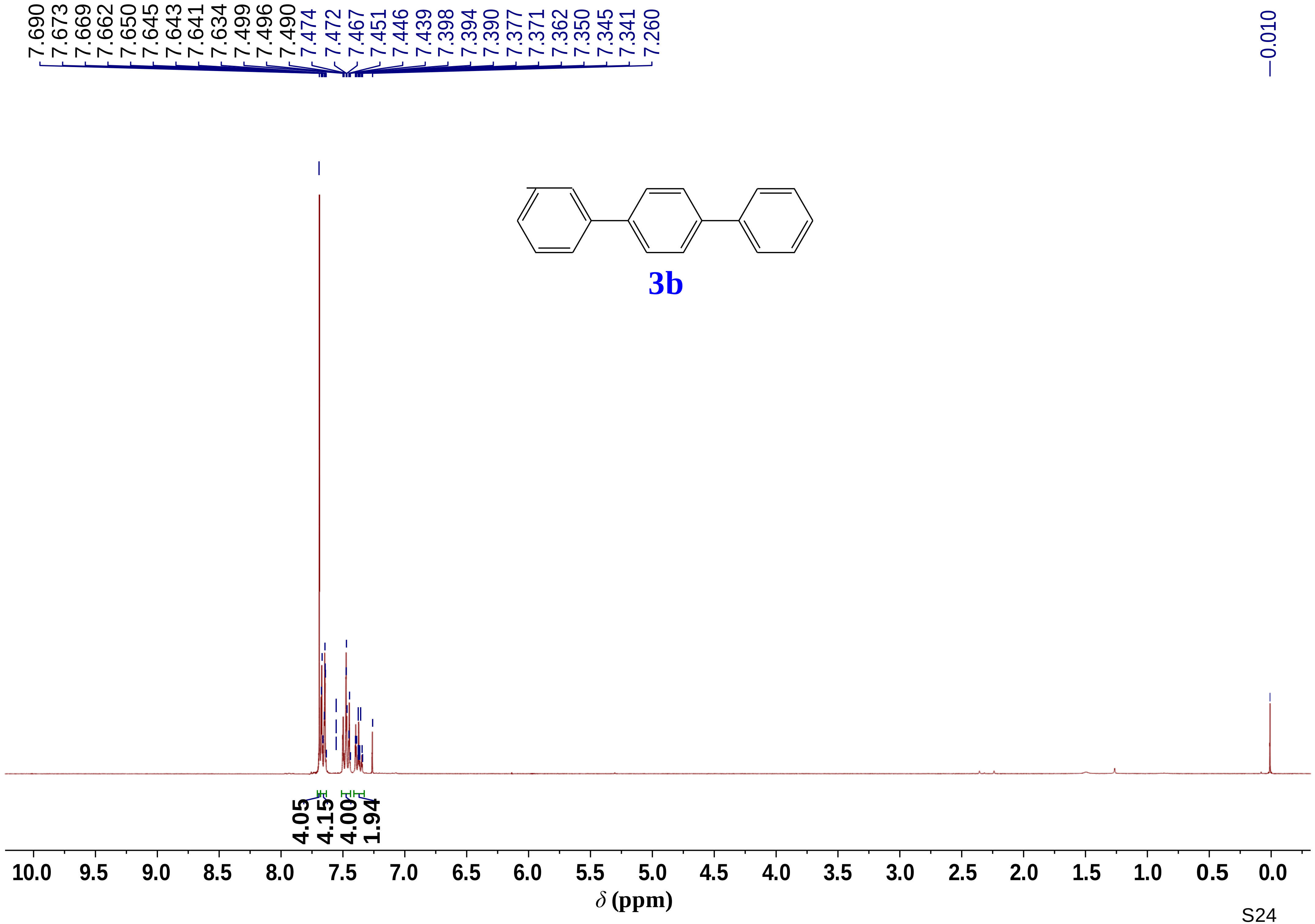
7.690
7.673
7.669
7.662
7.650
7.645
7.643
7.641
7.634
7.499
7.496
7.490
7.474
7.472
7.467
7.451
7.446
7.439
7.398
7.394
7.390
7.377
7.371
7.362
7.350
7.345
7.341
7.260
-0.010
3b
4.05
4.15
4.00
1.94
10.0 9.5 9.0 8.5 8.0 7.5 7.0 6.5 6.0 5.5 5.0 4.5 4.0 3.5 3.0 2.5 2.0 1.5 1.0 0.5 0.0
δ (ppm)

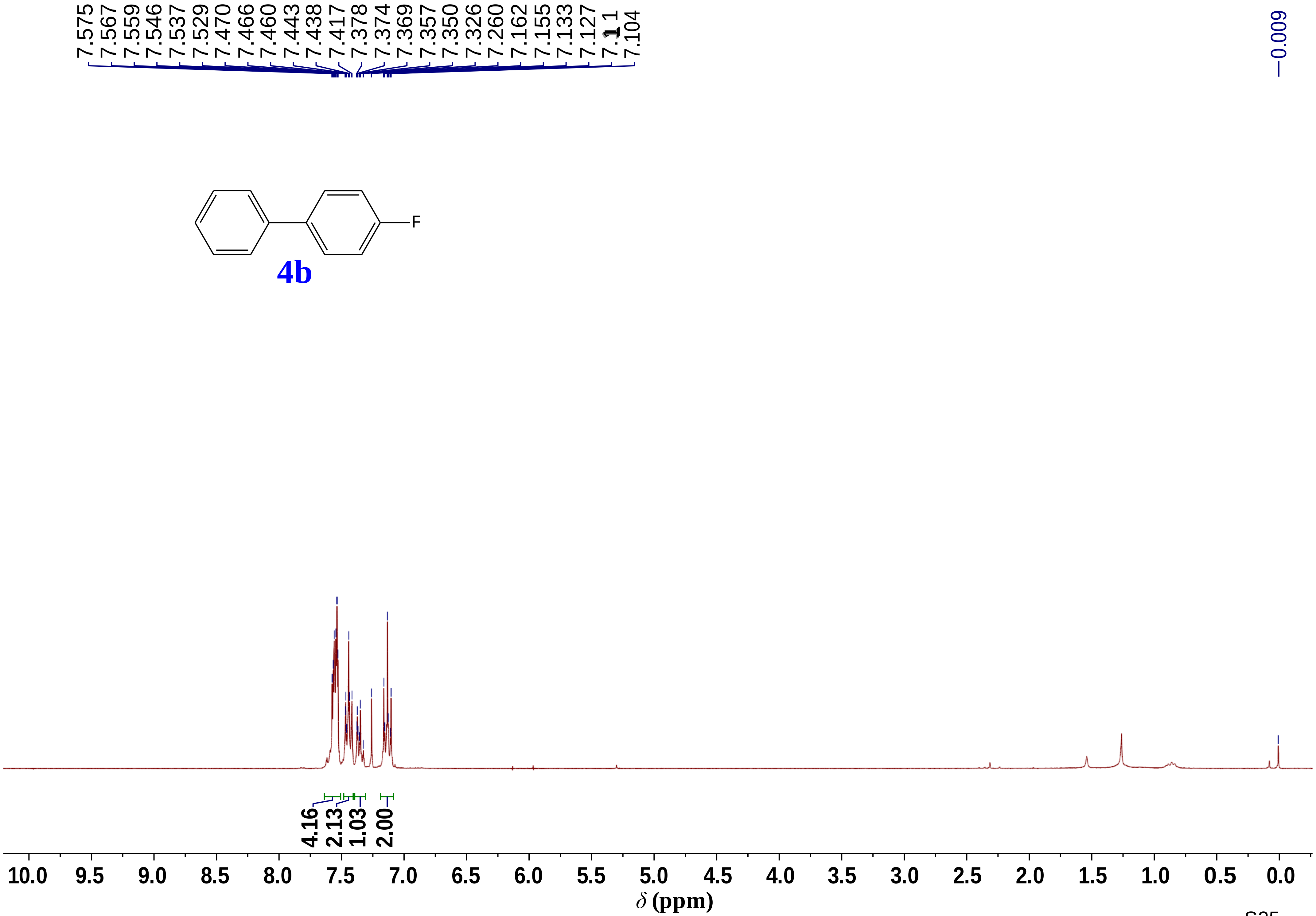
7.575
7.567
7.559
7.546
7.537
7.529
7.470
7.466
7.460
7.443
7.438
7.417
7.378
7.374
7.369
7.357
7.350
7.326
7.260
7.162
7.155
7.133
7.127
7.104
−0.009
F
4b
4.16
2.13
1.03
2.00
10.0
9.5
9.0
8.5
8.0
7.5
7.0
6.5
6.0
5.5
5.0
4.5
4.0
3.5
3.0
2.5
2.0
1.5
1.0
0.5
0.0
δ (ppm)

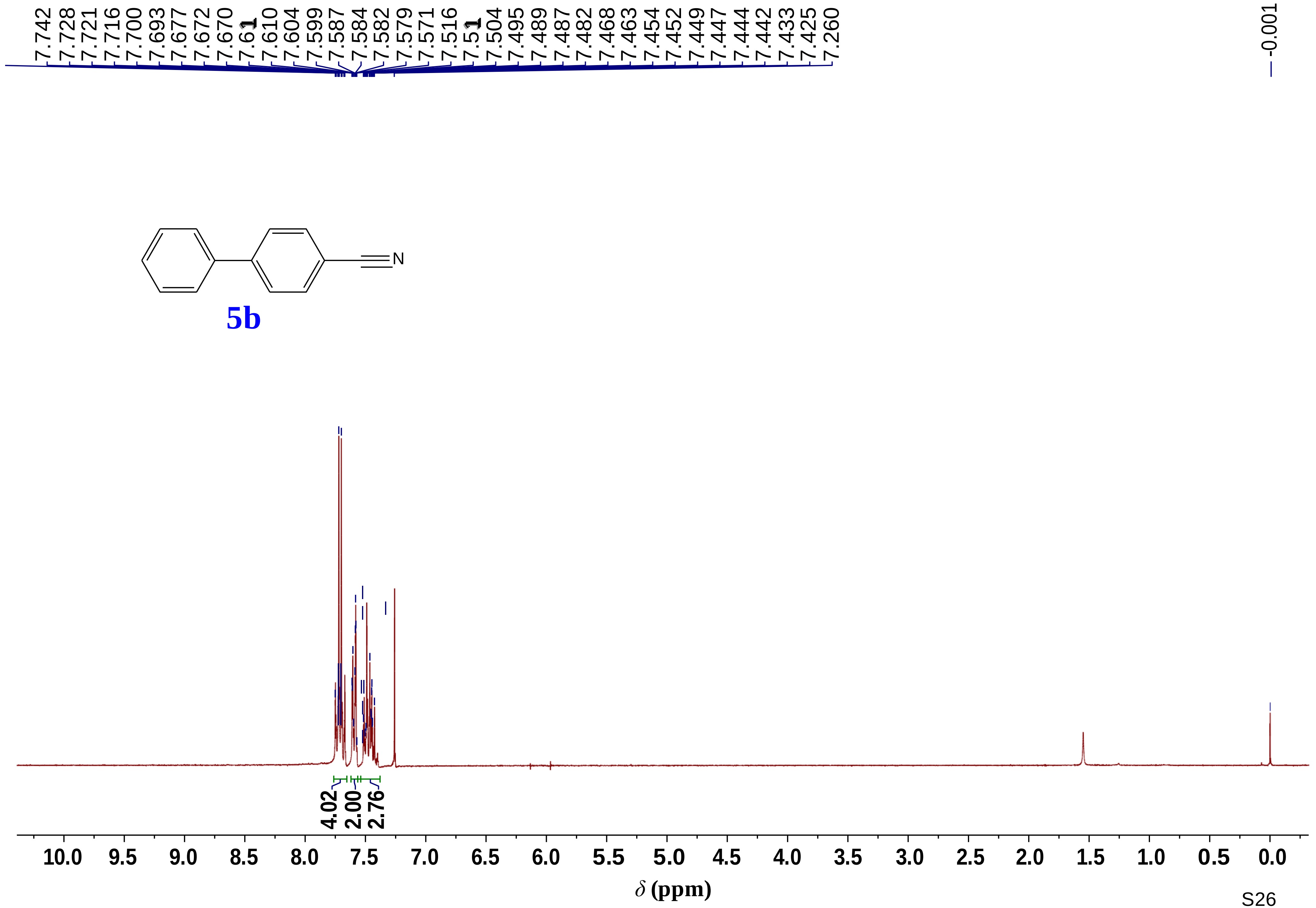
7.742
7.728
7.721
7.716
7.700
7.693
7.677
7.672
7.670
7.61[illegible]
7.610
7.604
7.599
7.587
7.584
7.582
7.579
7.571
7.516
7.51[illegible]
7.504
7.495
7.489
7.487
7.482
7.468
7.463
7.454
7.452
7.449
7.447
7.444
7.442
7.433
7.425
7.260
-0.001
5b
N
4.02
2.00
2.76
10.0
9.5
9.0
8.5
8.0
7.5
7.0
6.5
6.0
5.5
5.0
4.5
4.0
3.5
3.0
2.5
2.0
1.5
1.0
0.5
0.0
δ (ppm)

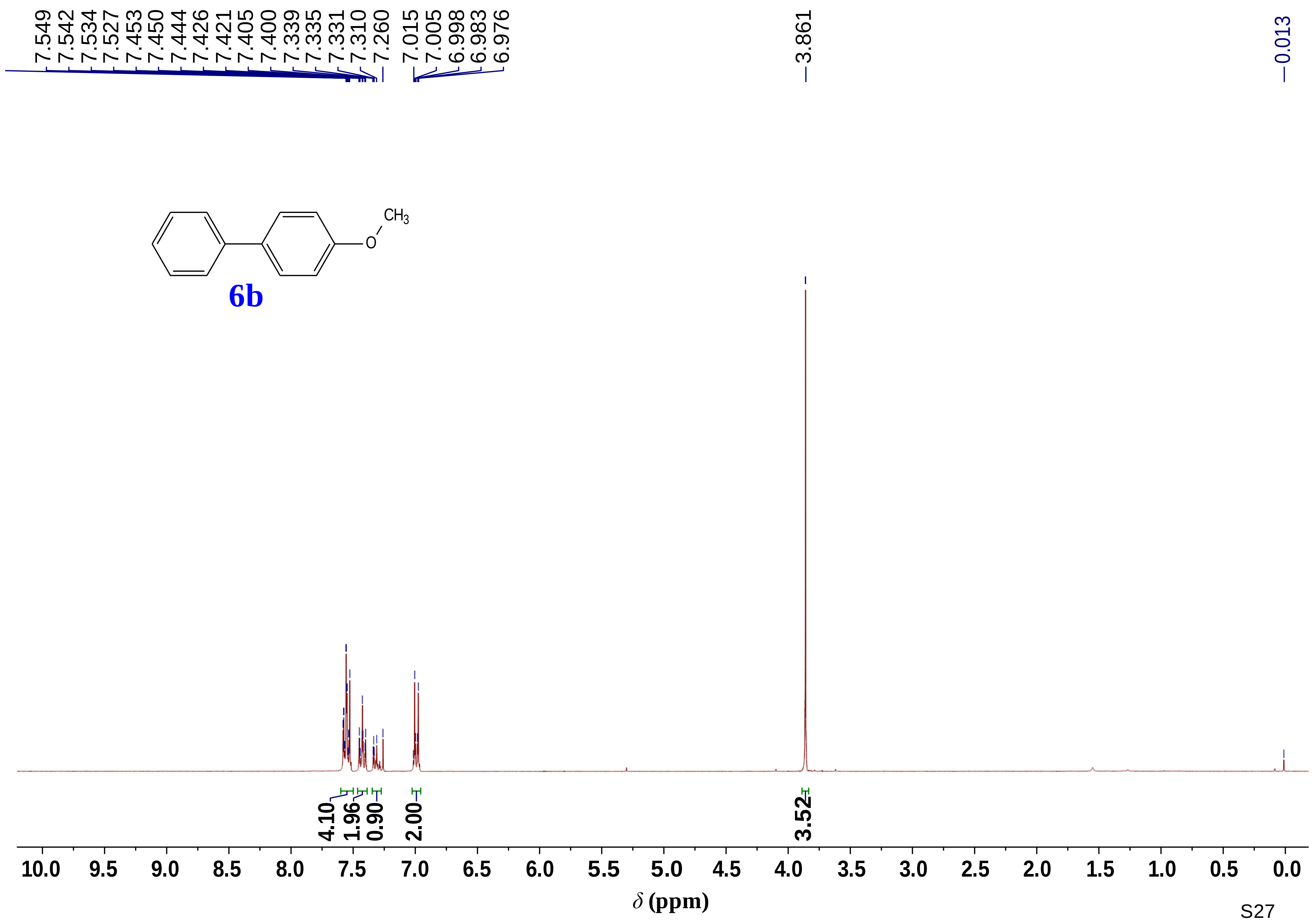
7.549
7.542
7.534
7.527
7.453
7.450
7.444
7.426
7.421
7.405
7.400
7.339
7.335
7.331
7.310
7.260
7.015
7.005
6.998
6.983
6.976
3.861
0.013
CH3
O
6b
4.10
1.96
0.90
2.00
3.52
10.0
9.5
9.0
8.5
8.0
7.5
7.0
6.5
6.0
5.5
5.0
4.5
4.0
3.5
3.0
2.5
2.0
1.5
1.0
0.5
0.0
δ (ppm)

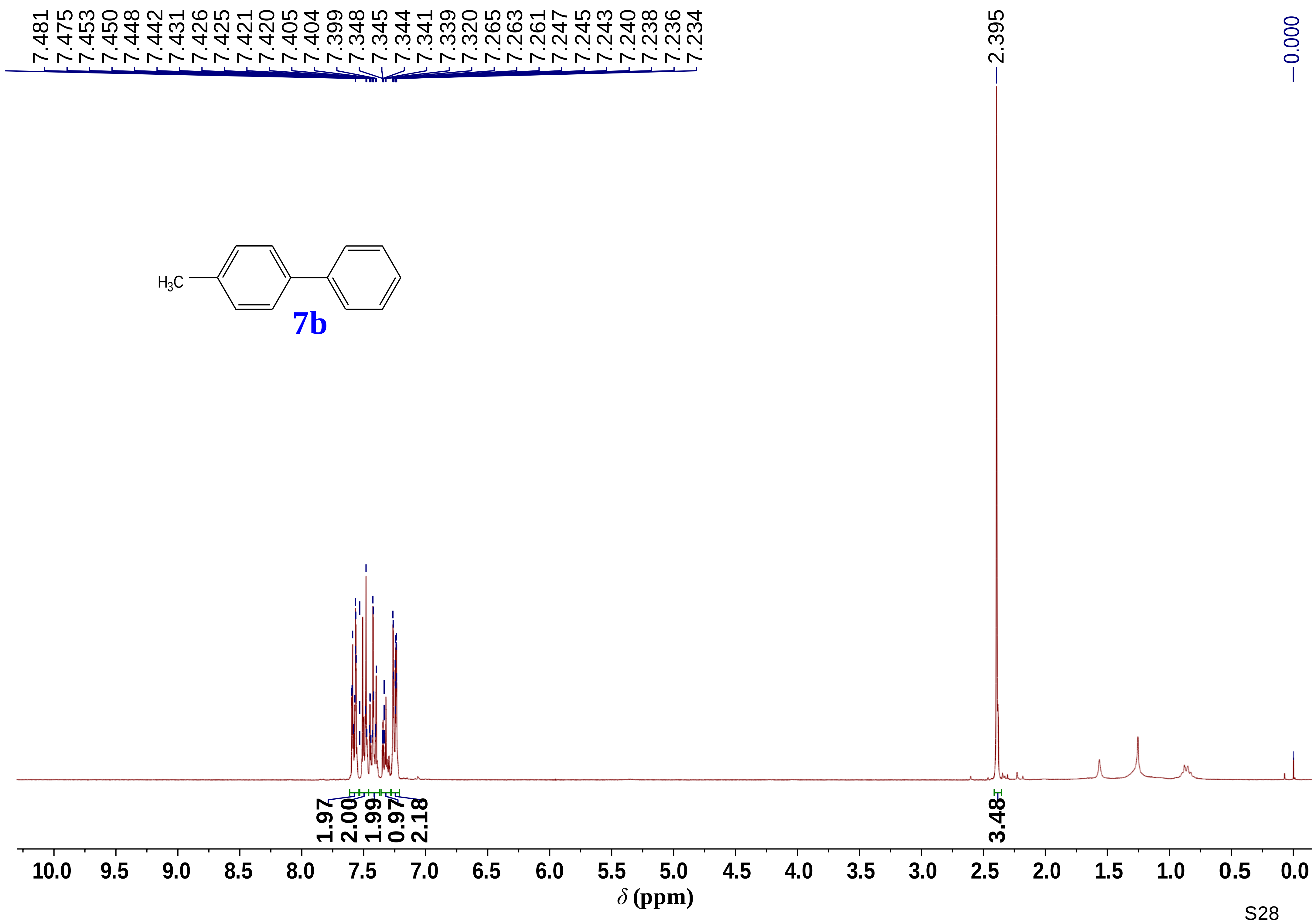
7.481
7.475
7.453
7.450
7.448
7.442
7.431
7.426
7.425
7.421
7.420
7.405
7.404
7.399
7.348
7.345
7.344
7.341
7.339
7.320
7.265
7.263
7.261
7.247
7.245
7.243
7.240
7.238
7.236
7.234
2.395
0.000
H_3C
7b
1.97
2.00
1.99
0.97
2.18
3.48
10.0 9.5 9.0 8.5 8.0 7.5 7.0 6.5 6.0 5.5 5.0 4.5 4.0 3.5 3.0 2.5 2.0 1.5 1.0 0.5 0.0
δ (ppm)

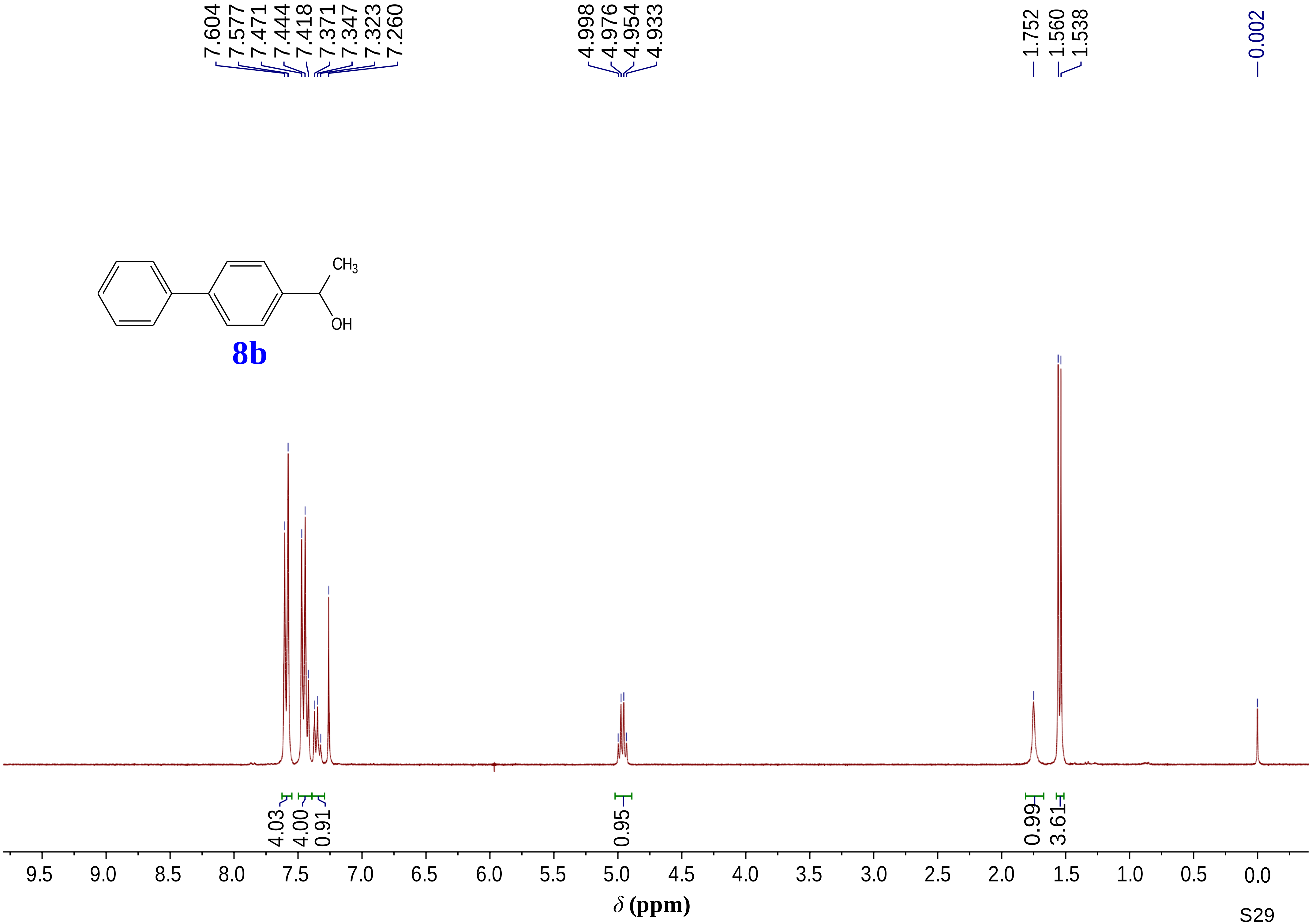
7.604
7.577
7.471
7.444
7.418
7.371
7.347
7.323
7.260
4.998
4.976
4.954
4.933
1.752
1.560
1.538
0.002
CH3
OH
8b
4.03
4.00
0.91
0.95
0.99
3.61
9.5 9.0 8.5 8.0 7.5 7.0 6.5 6.0 5.5 5.0 4.5 4.0 3.5 3.0 2.5 2.0 1.5 1.0 0.5 0.0
δ (ppm)

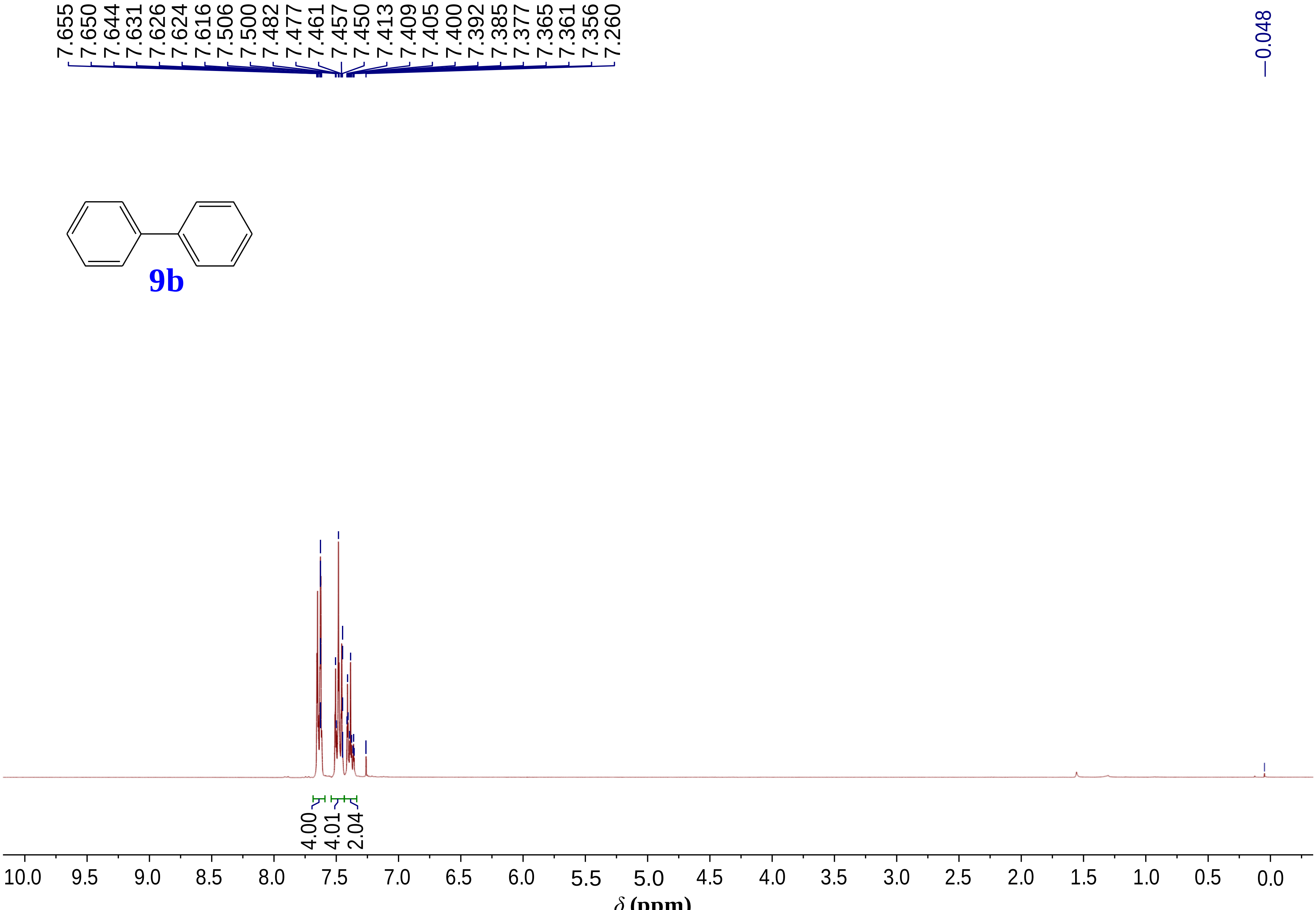
7.655
7.650
7.644
7.631
7.626
7.624
7.616
7.506
7.500
7.482
7.477
7.461
7.457
7.450
7.413
7.409
7.405
7.400
7.392
7.385
7.377
7.365
7.361
7.356
7.260
0.048
4.00
4.01
2.04
10.0 9.5 9.0 8.5 8.0 7.5 7.0 6.5 6.0 5.5 5.0 4.5 4.0 3.5 3.0 2.5 2.0 1.5 1.0 0.5 0.0
δ (ppm)

9b
